# Adversarial Machine Learning in Industrial IoT: A Systematic Review of Attack Realism, Defense Trade-Offs, and Deployment Gaps

**DOI:** 10.3390/s26165098

**Published:** 2026-08-11

**Authors:** Abeer Alsaidlani, Muhammad Rashid, Malak Aljabri

**Affiliations:** Department of Computer and Networks Engineering, College of Computing, Umm Al-Qura University, Makkah 24382, Saudi Arabia; mfelahi@uqu.edu.sa (M.R.); mssjabri@uqu.edu.sa (M.A.)

**Keywords:** adversarial attacks, Industrial Internet of Things, machine learning, data protection, industrial security

## Abstract

Modern Industrial Internet of Things (IIoT) integrates machine learning models for monitoring and control. However, they remain vulnerable to adversarial machine learning (AML) attacks, where an adversary adds small changes to the input data. These small changes degrade model quality, reduce accuracy, and can ultimately compromise the safety and security of the entire system. AML research in IIoT often focuses on individual attack types, defense methods, and datasets. Existing reviews lack a unified quantitative and system-level perspective. Therefore, a systematic literature review (SLR) is needed to provide a holistic analysis of existing attacks, defenses, and databases. This SLR analyzes 50 research articles to provide a holistic view of AML threats in IIoT systems and identifies seven distinct attack types: gradient-based perturbations, GAN-generated samples, poisoning attacks, reinforcement learning-based (RL) strategies, saliency-based feature manipulation, false data injection, and hybrid approaches. To illustrate the range of observed impacts, selected studies report the following degradation examples: saliency-based attacks cause accuracy reductions of 6–11 percentage points; iterative gradient attacks reduce accuracy from 95–99% to 30–40% in SIEM systems; and RL-based attacks reduce detection rates from 100% to 0% in rule-based IDS settings. In addition to the analysis of attack types, this SLR also evaluates current defense methods to protect IIoT systems. It has been observed that existing defense mechanisms lack generalization and require high computational resources. Moreover, the testing is performed under simplified threat models. The analysis of datasets further shows a clear gap between realistic industrial benchmarks (such as SWaT, WADI, and NSL-KDD) and synthetic datasets used for controlled experiments. By connecting attack behavior, defense performance, dataset characteristics, and system-level effects, this SLR identifies the key research gaps that must be addressed in future work.

## 1. Introduction

**Figure 1:**
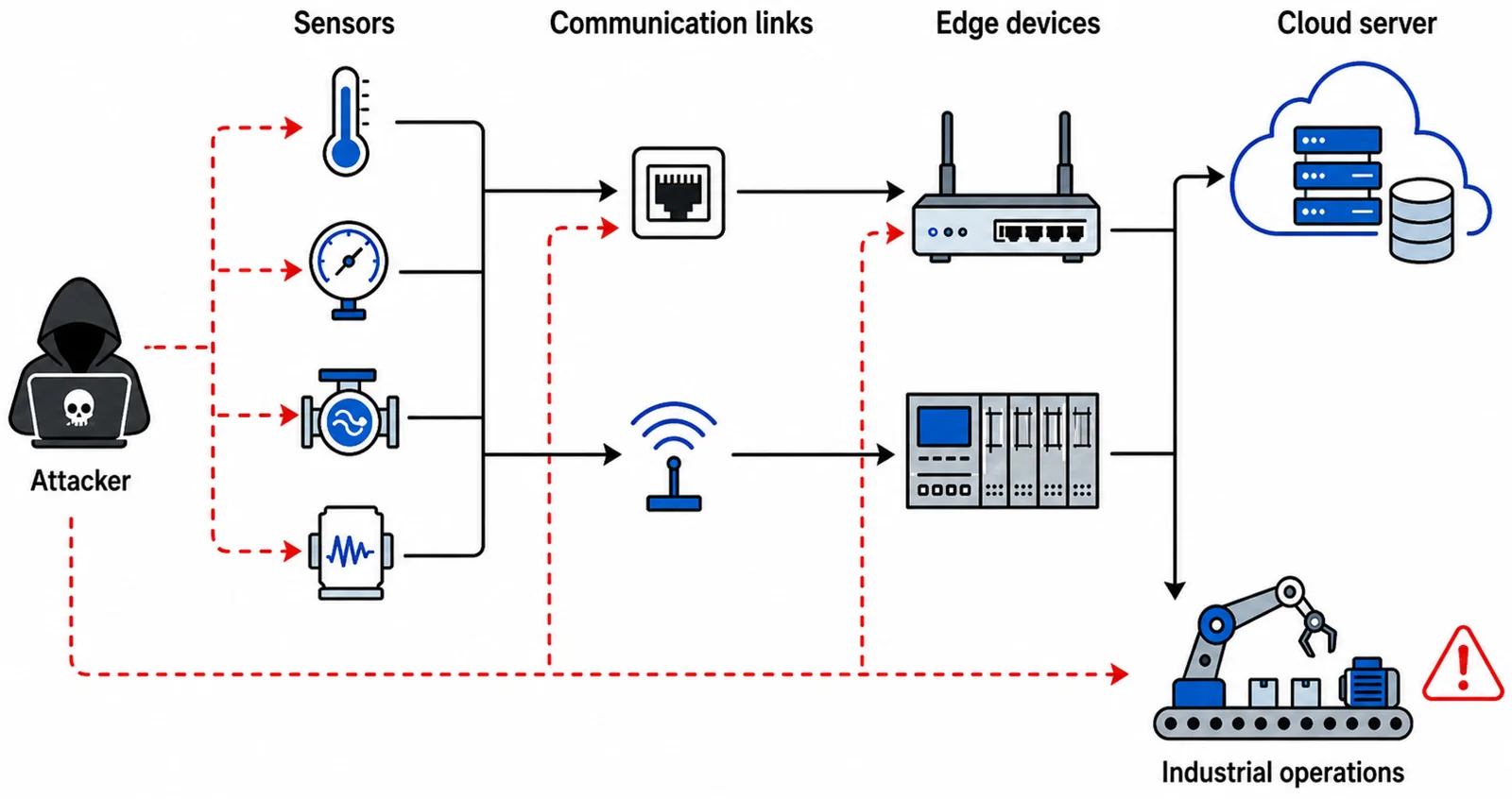
IIoT setup with connected devices, a cloud server, and an attacker. Note: Black arrows indicate the normal communication and data flow, whereas red dashed arrows indicate the attack path.

The Industrial Internet of Things (IIoT) systems integrate sensors, controllers, communication networks, and computational platforms. These components allow continuous data collection in real-time and provide adaptive control in industrial systems [1,2]. However, this interconnected nature also exposes IIoT systems to multiple attack surfaces [3,4,5]. Figure 1 shows how different components (sensors, communication links, edge devices) can be attacked to disrupt industrial operations [6].

**Figure 2:**
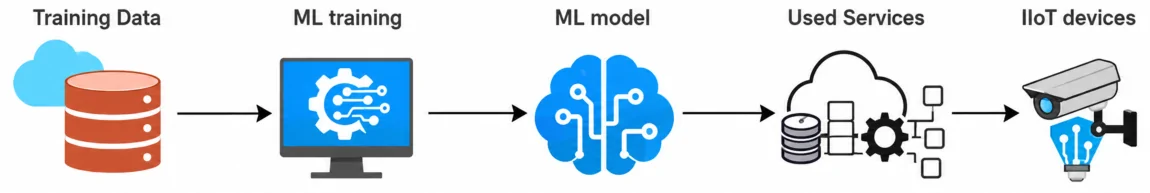
Overview of a simplified IIoT ML workflow.

With the continuous increase in IIoT interconnectivity, machine learning (ML) and deep learning (DL) models have become essential [5]. Figure 2 presents a typical ML/DL pipeline and its interaction with IIoT devices. These models process sensor data, detect anomalies, and support automated decision-making. The process begins with collecting training data, which is used to build the ML model. Once the model is trained, it supports IIoT devices and services such as monitoring, prediction, and automated control.

While ML and DL techniques play an important role in intelligent automation, they also introduce new security risks [7,8]. Each interaction point in the ML/DL pipeline serves as a potential attack surface, allowing adversaries to manipulate data, models, and overall system behavior [9]. The problem becomes more serious in safety-critical environments [10,11]. In these environments, small input changes can trigger false control actions [12]. These false operations create risks for both operational reliability and physical safety. Defenses against these threats require significant computational resources. However, IIoT devices often have limited processing power. Therefore, defenses are harder to implement. As a result, attacks become more harmful in real IIoT environments [13].

In IIoT systems, ML and DL models are not used for a single purpose. They support several practical tasks, and each task creates a different type of attack surface. For example, anomaly and intrusion detection models (such as LSTM- or CNN-based detectors) monitor sensor readings and network traffic. Fault diagnosis and condition monitoring models process signals such as vibration or hydraulic sensor data [14,15].

State estimation and false-data detection models are used with power-grid measurements [16]. Reinforcement learning-based controllers are used to guide actuator behavior in closed-loop industrial processes [17]. Because these tasks operate in different settings, the meaning of a “realistic” adversarial perturbation also changes. For anomaly detection, the manipulated data should still preserve temporal consistency. For fault diagnosis, the perturbation should remain within physically plausible sensor ranges. For closed-loop control, the attack cannot be judged only from a static input sample; it needs to be evaluated within the feedback loop. This diversity makes the IIoT attack surface more complex than domains such as general image classification, where inputs are usually less constrained.

Beyond ML-focused attack surfaces, other cyber-security studies show that adversarial behavior also appears at the traffic and protocol layers. ADVoIP demonstrates this by analyzing encrypted VoIP streams. Attackers hide traffic through padding, shifting, and splitting operations, while defenders try to detect these manipulations [18]. Game-theoretic models employ a switched Stackelberg game to study DoS-affected switched systems [19]. APT (Advanced Persistent Threat) defense has also been framed as a rivalry game. It proposes learning mechanisms based on adversarial bandits and deep reinforcement learning [20]. Overall, these works show that securing networked and cyber-physical systems requires understanding traffic manipulation, strategic attacker behavior, and how defensive decisions evolve over time.

### 1.1. Motivation for a Systematic Literature Review

- **Lack of a Holistic View:** Existing studies on adversarial attacks in IIoT target different application domains [21,22]. Therefore, it is always difficult to compare threats across systems. Consequently, a systematic literature review (SLR) can unify these findings and compare methods in terms of common performance parameters.
- **Inconsistent Evaluation of Defenses:** Existing defense methods are evaluated under different datasets and attack models. This inconsistency limits their applicability in real industrial settings. An SLR can compare these defenses and identify which approaches are most suitable [23].
- **Lack of Standardized Benchmarks:** Existing datasets exhibit different characteristics in terms of fidelity, format, and domain focus. Therefore, it is difficult to compare results or generalize findings across research studies [24]. Moreover, IIoT-specific benchmarks are missing. Consequently, a systematic review is needed to explore how datasets are used. It should also highlight inconsistencies and identify gaps that must be filled to develop reliable AML solutions for IIoT.

### 1.2. State-of-the-Art Reviews and Limitations

Table 1 summarizes surveys and analytical studies on AML attacks and defenses in IoT and IIoT environments. Some works, such as [25], use systematic review methods to evaluate different techniques, while others, including [26], provide experimental analysis. General AML surveys [27,28] present broad taxonomies of attacks and defenses but do not consider the operational constraints of industrial systems. Similarly, IoT-focused reviews [29] describe explanations of adversarial concepts but do not fully address the constraints and deployment challenges of IIoT systems. In addition to the aforementioned surveys, the works in [22,30,31] target AML techniques across intrusion detection, medical, and industrial settings, respectively. They analyze ML-based models and propose taxonomies that integrate security and privacy. A system-level analysis of attacks and defense methods is presented in [4]. It highlights the need for standardized testing and scalable defense methods. Industry-oriented assessments [32] show that many organizations are vulnerable to ML-specific security risks. Practitioner interviews show that teams often lack proper tools. Moreover, there is limited awareness of ML-related threats, and well-defined procedures are uncommon.

Table 1 reveals that the prior work has not brought together attack types, defenses, dataset practices, and threat-model realism into a single IIoT-focused framework. They share several limitations: (a) only one provides a fully systematic assessment, (b) most lack a holistic evaluation of attacks, defenses, and datasets, and (c) there are no standardized protocols or unified robustness metrics. Moreover, many studies in Table 1 offer useful taxonomies and conceptual overviews, but they remain disconnected from real IIoT operational needs. As a result, this SLR goes beyond simply listing adversarial ML studies. First, it systematically evaluates how realistic the assumed threat models are across seven attack categories, distinguishing between white-box, gray-box, black-box, and process-aware settings in IIoT environments. Second, it examines whether commonly used datasets are actually suitable for IIoT, asking if they capture physical process behavior, sensor–actuator relationships, realistic protocols, and deployment conditions. Third, it draws attention to an under-explored compound threat in which false data injection attacks interact with training-pipeline poisoning, creating a cross-phase attack path that prior IIoT adversarial ML reviews have not clearly described. By bringing these aspects together, the review offers a more deployment-aware view of adversarial robustness in IIoT systems.

In addition to the review articles listed in Table 1, wireless-communication surveys have also examined ML security, robustness, and generalization from different perspectives. Some focus directly on adversarial attacks against DNN-based wireless applications [33], including over-the-air perturbations that may be affected by channel conditions, receiver filtering, fading, noise, and other physical-layer effects. Other surveys discuss distributed ML [34], domain generalization [35], and ML-enabled 6G systems [36], covering topics such as federated learning, MAML, GANs, spectrum management, channel estimation, beamforming, and edge/cloud wireless services. These works are relevant because many IIoT deployments use wireless links, and adversarial perturbations in such settings may be altered before reaching the target model. However, the scope of the present review is different. Wireless AML surveys mainly focus on signal-level or communication-layer tasks, whereas this review focuses on adversarial ML in IIoT and industrial cyber–physical systems, including anomaly and intrusion detection, fault diagnosis, state estimation, false-data detection, and control-oriented learning. These IIoT tasks introduce additional constraints related to physical plausibility, temporal consistency, process safety, and closed-loop operation. Therefore, the incremental contribution of this review is to organize AML attacks and defenses around IIoT-specific datasets, threat models, attack surfaces, and operational constraints, while identifying wireless-channel-aware AML evaluation as an important direction for future IIoT research.

### 1.3. Research Questions

The limitations identified in existing review articles have been addressed in this SLR by formulating the following research questions.

- **RQ1: What are major AML attacks in IIoT environments, and how do they affect system performance?**
- **RQ2: What defense mechanisms have been proposed, and how effective are they in IIoT settings?**
- **RQ3: How do datasets differ across studies, and what are the consequences of these differences?**
- **RQ4: What open challenges remain and what are the probable future research directions?**

### 1.4. SLR Framework: Novelty and Contributions

**Figure 3:**
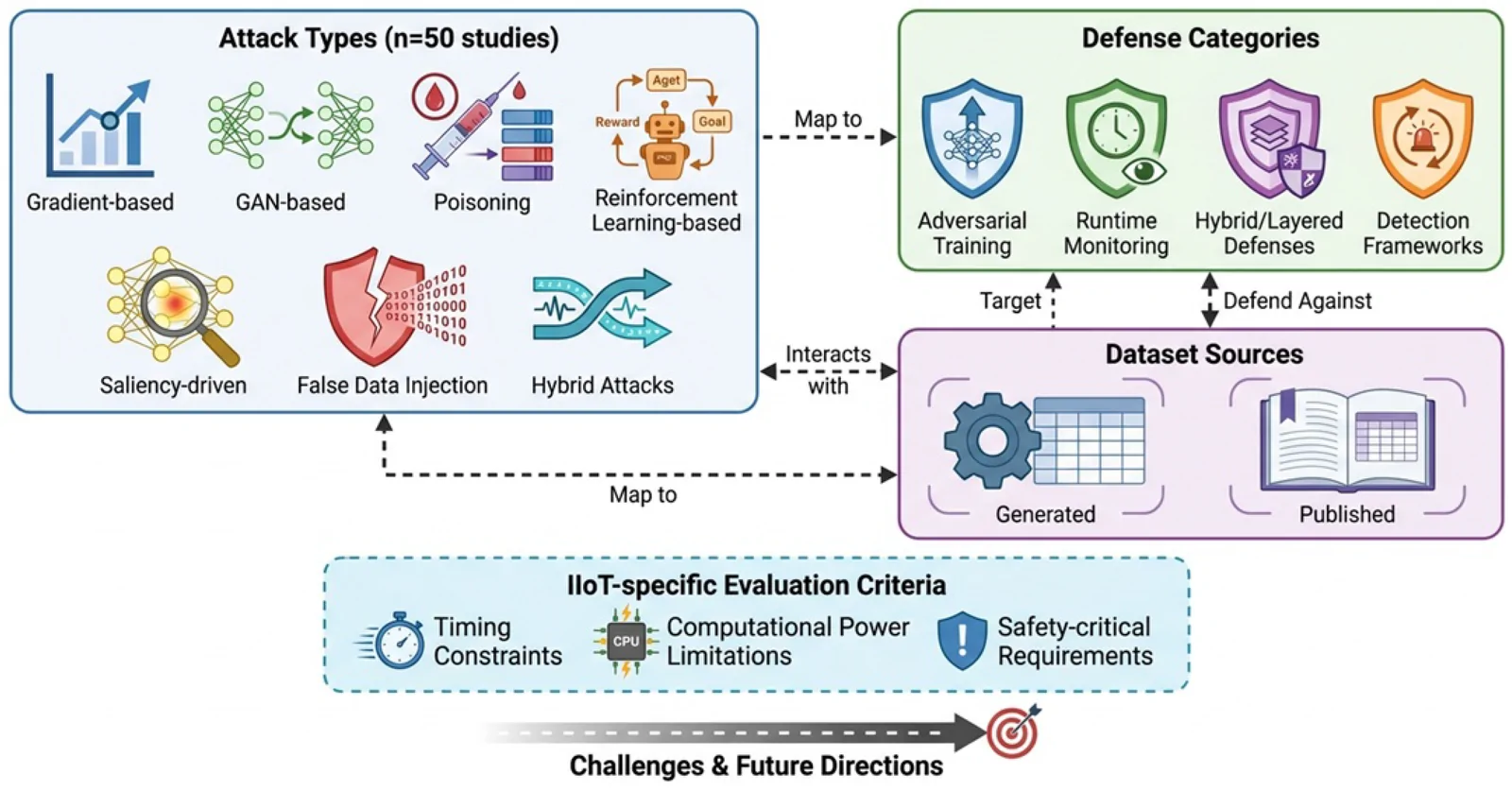
Overview of the SLR structure, summarizing attack types, defense strategies, dataset sources, and IIoT-specific evaluation criteria.

A holistic overview of the SLR framework is shown in Figure 3. The SLR categorizes the selected studies (Total = 50) into seven major attack types. These types are gradient-based, GAN-based, poisoning, reinforcement learning-based, saliency-driven, false data injection, and hybrid attacks. Moreover, four defense categories are identified: adversarial training, runtime monitoring, hybrid/layered defenses, and detection frameworks. It also classifies datasets into generated and published sources. Unlike generic AML reviews [27,28], this SLR is mainly targeting IIoT environments. Therefore, timing constraints, computational power limitations, and safety-critical requirements have been evaluated for the selected studies. In other words, this SLR explores attack–defense–dataset interdependencies and provides a holistic understanding of the domain. Finally, based on observations, the challenges have been identified and probable future directions have been proposed.

## 2. Literature Review Methodology

Our methodology is mainly based on the recommendations provided in [37]. A similar methodology was adopted in previous reviews on EdgeML and TinyML deployment [38,39], ML in underwater acoustic communication systems [40], and bridge structural health monitoring systems [41,42]. The major steps in the methodology are: (a) identification of categories and (b) developing a review protocol for selecting relevant articles to answer formulated research questions. Consequently, Section 2.1 presents the background of these categories, while Section 2.2 explains the steps of the review protocol in detail.

### 2.1. Background on Categories

The research articles were organized into three categories. This allows a coherent evaluation of AML research within IIoT environments. Before presenting the categorization, we clarify four terms that are used precisely throughout this review. An **attack** is a specific adversarial method (e.g., FGSM, JSMA) used to manipulate data or models, whereas a **threat** describes the broader adversarial capability or scenario independent of any particular attack. **Detection** identifies adversarial or anomalous inputs without necessarily addressing the underlying vulnerability. **Classification** refers to assigning labels to inputs and is distinct from adversarial detection. **Defense** encompasses detection and mitigation strategies, including adversarial training, runtime monitoring, hybrid approaches, and detection-based frameworks, that reduce vulnerability to a given threat.

#### 2.1.1. Adversarial Attacks in IIoT

**Figure 4:**
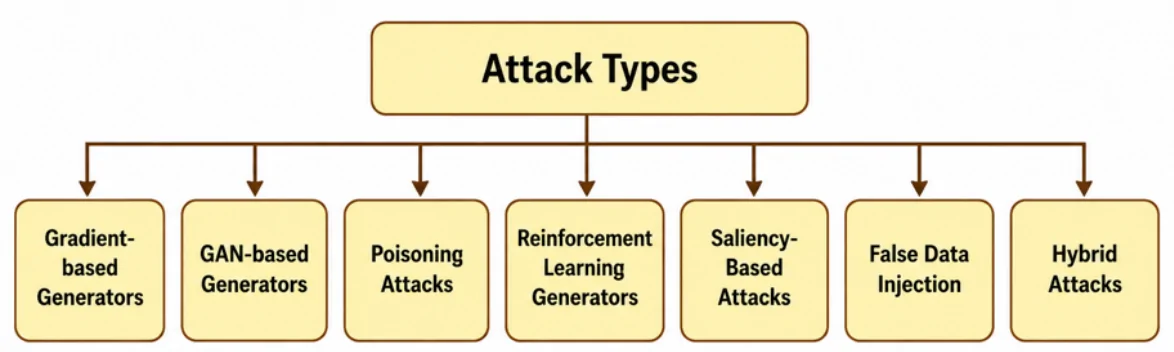
Classification of adversarial attack types in IIoT.

Figure 4 shows that the selected studies can be classified into distinct attack types: gradient-based attacks, GAN-generated perturbations, poisoning attacks, RL-driven adversarial strategies, saliency-guided perturbations, false data injection attacks (FDIA), and hybrid or composite generation approaches.

#### 2.1.2. Defense Mechanisms in IIoT

**Figure 5:**
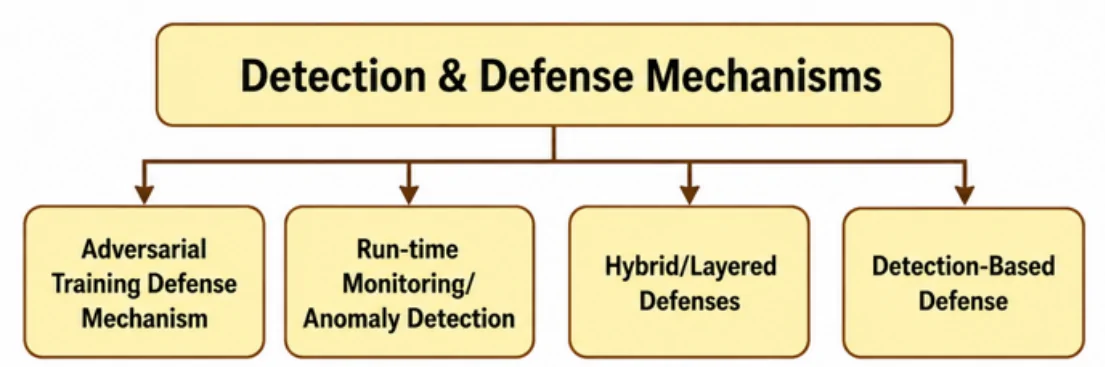
Main categories of detection and defense mechanisms against adversarial attacks in IIoT environments.

Figure 5 shows existing defense methods can be organized into adversarial training-based defenses, runtime monitoring and anomaly-detection frameworks, hybrid or layered security architectures, and ML-based detection frameworks.

#### 2.1.3. Dataset Utilization and Benchmarking in IIoT

Section 6 organizes the selected research studies based on datasets used for training, testing, or validating attacks and defenses in IIoT environments. The analysis includes IIoT-specific datasets, SCADA datasets, general cybersecurity benchmarks, and artificially generated datasets used for adversarial experimentation. This category highlights inconsistencies in dataset selection processes and explores why the IIoT-specific benchmarks are still missing. It also emphasizes the need for standardized datasets to perform a fair performance evaluation of AML research in IIoT environments [24].

### 2.2. Review Protocol Development

Developing a review protocol is essential in a typical literature review process. It involves selection/rejection criteria (Section 2.2.1) and search process (Section 2.2.2).

#### 2.2.1. Criteria for Selection and Rejection

**Figure 6:**
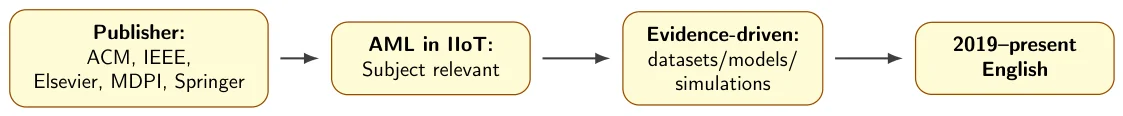
Inclusion criteria for the selection of research articles.

**Figure 7:**
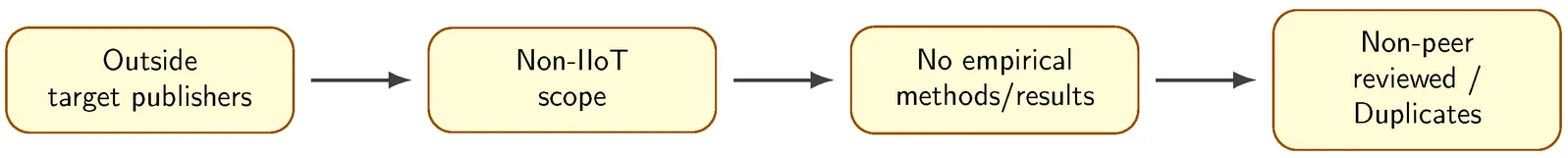
Exclusion criteria for the rejection of research articles.

The selection/rejection criteria are summarized in Table 2 and visually shown in Figure 6 and Figure 7. The research articles have been selected from multiple databases such as ACM, IEEE, Elsevier, MDPI, and Springer. The motivation behind the selection of these databases is their wide coverage of the target research domain. A total of 50 studies have been selected. All the selected studies are based on strong experimentation. Journal publications were preferred over conference papers to ensure stronger experimental validation.

#### 2.2.2. Search Process

**Figure 8:**
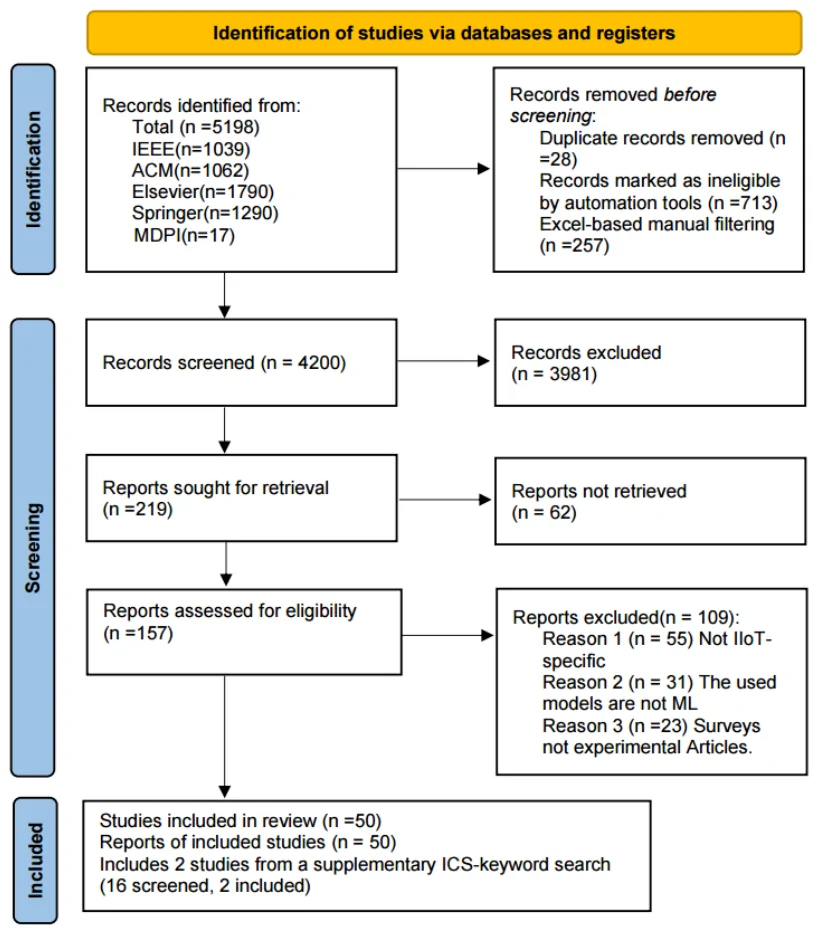
PRISMA Flow Diagram.

**Figure 9:**
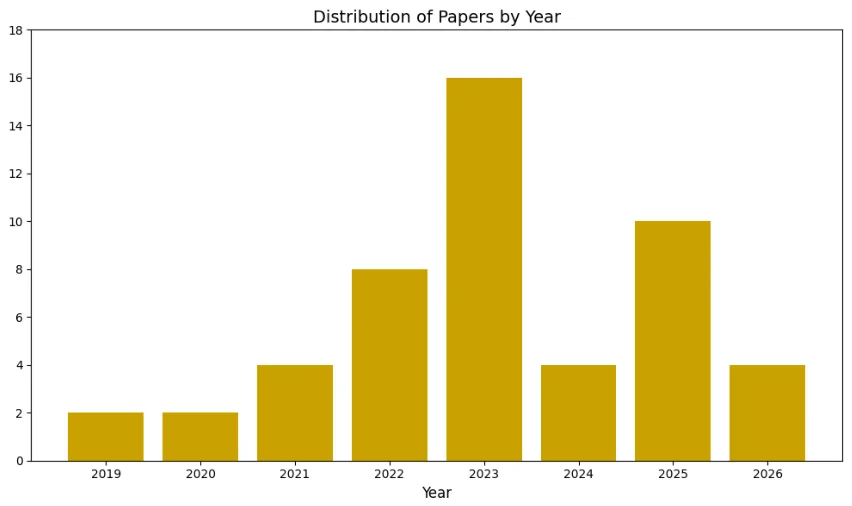
Distribution of selected articles over the years.

Using the query ((“adversarial machine learning” OR “adversarial attack” OR “adversarial defense”) AND (“Industrial Internet of Things” OR “IIoT” OR “industrial IoT” OR “ICS”) AND (“machine learning” OR “ML”)) and limiting the period to 2019–2026, a total of 5198 publications have been found from the selected databases.After title and abstract screening, 157 candidate studies remained. The remaining articles have been reviewed in the light of Table 2. An additional supplementary search was conducted using the keyword “ICS” more directly with adversarial machine learning terms. This search followed the same databases and eligibility criteria and produced 16 candidate studies. Two additional studies met all inclusion criteria and were not captured in the original search. One was added under hybrid attacks, and the other under runtime anomaly detection defenses. Accordingly, the final review includes 50 studies. More details on the identified studies from the selected databases and the number of excluded studies before and after screening for each stage are presented in Figure 8. In the end, 50 research articles were selected. Figure 9 presents the distribution of selected articles by publication year.

## 3. Classification Results

Table 3 classifies selected studies in terms of three predefined categories. The first category focuses on adversarial attack types. The major attack types are: gradient-based, GAN-generated, RL, hybrid, false data injection, poisoning, and saliency-based attacks. In total, 28 articles addressed these categories. The second category highlights defense mechanisms. It includes adversarial training, detection-based approaches, runtime monitoring, and hybrid defenses. 35 studies proposed such techniques. The final category examines data sources, distinguishing between public datasets and generated datasets. This dimension emphasizes the realism of experiments across all reviewed articles. Within the selected studies analyzed in Table 3, some perform both attack generation and corresponding defense mechanisms. Others focus either on attack development or on defense strategies. Finally, all studies provide the datasets for experimentation.

The reviewed literature reveals seven types of adversarial attack mechanisms summarized in Table 4. Each employs different techniques and targets specific stages of IIoT and related ML systems.

- Gradient-based attacks such as FGSM, PGD, and the CW attack exploit model gradients to craft small perturbations that mislead ML models during inference.
- In contrast, GAN-generated attacks produce highly realistic, semantically meaningful adversarial samples that closely resemble normal data. Other strategies include reinforcement learning-generated attacks that adaptively craft sequential perturbations in dynamic IIoT or industrial control settings, and FDIA, which compromise sensor readings to disrupt automated control systems and data-driven detectors. As far as poisoning attacks are concerned, they corrupt the training process by injecting malicious samples.
- Saliency-based attacks target the most influential features to enhance stealth.
- Hybrid-generated attacks combine multiple attack methods to enhance effectiveness.

In response to these evolving threats, existing research categorizes defense mechanisms into four types. Table 5 summarizes these defense categories with representative examples illustrating their practical implementation.

Before turning to the study-specific results discussed in the following sections, it is useful to first characterize each attack and defense category at the level of general principles, as summarized in Table 4 and Table 5. Among attack mechanisms, gradient- and saliency-based methods are computationally efficient but depend on strong model access; GAN- and RL-based methods trade higher computational cost for greater realism or adaptivity; poisoning and FDIA target different pipeline stages and therefore presuppose fundamentally different attacker capabilities; and hybrid attacks combine these trade-offs rather than resolving them. Among defense mechanisms, adversarial training and detection-based methods are the most computationally accessible but the least robust to novel or adaptive threats, while runtime monitoring and hybrid defenses trade higher overhead for stronger and more comprehensive protection. These general characteristics, rather than the results of any single study, motivate the mechanism-plus-stage taxonomy adopted in this review and provide the baseline against which the per-study findings in the following subsections should be interpreted.

## 4. Adversarial Attack Taxonomy for IIoT

The seven attack categories in this SLR are derived through an inductive analysis of 50 selected studies. Instead of starting with a predefined taxonomy, we carefully examine each selected study and group attacks based on their common characteristics, such as how they operate, the type of data they target, the stage of the machine learning pipeline they affect, and their overall objective, as summarized in Table 4. Each category therefore represents a distinct and practical way in which IIoT systems can be attacked. For example, gradient-based and saliency-based attacks both rely on knowledge of the internal model, but they differ in their approach: gradient-based attacks make broad changes across the input, while saliency-based attacks carefully target only the most important features to remain more subtle. Similarly, GAN-based and reinforcement learning-based attacks both use learning techniques, but GANs focus on generating realistic-looking adversarial data, whereas reinforcement learning methods learn how to attack step by step through interaction with the system. Poisoning and false data injection (FDIA) attacks both involve manipulation of data, but they occur at different stages—poisoning corrupts the training data, while FDIA alters real-time sensor data during system operation. Hybrid attacks combine multiple attack strategies to increase their effectiveness and flexibility. Overall, this taxonomy is grounded directly in patterns observed in the reviewed studies, rather than being imposed from existing frameworks, which makes it more representative of current research trends. The following subsections further explain each category and compare the findings from different studies in a clear and structured manner. Existing AML taxonomies surveyed in Table 1 organize attacks using different principles: some link attack mechanisms directly to defense strategies [27], others categorize adversarial strategies into distinct types without a unifying access-model or stage-based structure [28], and others integrate security, privacy, and reliability considerations into a single taxonomy [31]. However, as Table 1 also indicates, these works are consistently noted to lack a holistic, IIoT-specific perspective and standardized benchmarks that jointly integrate attacks, defenses, and datasets. Rather than adopting any single one of these organizing principles, this review uses an inductive, mechanism-plus-stage classification: attacks are grouped by their underlying generation mechanism (gradient-based, GAN-based, RL-based) and by the pipeline stage and data modality they target, as distinguished for training-time poisoning versus inference-time FDIA in Section 4.6. This approach is preferable for IIoT because it does not treat access-model or perturbation-type alone as the primary organizing axis; instead, it is explicitly cross-validated against the access-model distribution reported in Section 4.8 and the domain/modality distribution reported in Section 4.9—the joint attack–defense–dataset integration that Table 1 identifies as missing from prior taxonomies.

### 4.1. Gradient-Based Generators

The studies in this category use several gradient-based attack methods that differ mainly in how they compute and apply perturbations. FGSM and BIM both move in the direction of the sign of the loss gradient, typically under an $L_{\infty }$ constraint, but FGSM does this in a single step while BIM applies smaller updates over multiple iterations. PGD builds on this iterative idea by adding a projection step after each update to keep the perturbation within a specified norm ball, which usually makes the attack stronger but also more computationally expensive. In contrast, the CW attack frames perturbation generation as an optimization problem that explicitly minimizes the perturbation size (under L1,L2, or $L_{\infty }$ norms) while still forcing misclassifications, and often produces smaller, more subtle perturbations than FGSM, BIM, or PGD.

Table 6 shows that gradient-based attacks are the most widely used. Even simple one-step attacks (such as FGSM) can cause a comprehensive degradation with minimal changes. For example, the work in [53] drops 20–22.5% precision, recall, and F1-score when only 10% of input features are perturbed. Similarly, the work in [61] shows LSTM-based SIEM accuracy falls from 95–99% to 30–40%. Iterative attacks such as PGD intensify this impact. In [46], accuracy drops from 80–90% to 65–75% under $L_{\infty }$-bounded perturbations. The work in [45] represents almost a complete model failure. FGSM and BIM reduce accuracy from approximately 98.7% to 0.03%.

More recent attack methods (such as VAASI [43]) further increase attack effectiveness. VAASI achieves up to 85.5% success rates and maintains strong classification performance (Precision = 0.974, Recall = 0.972, F1 = 0.973). It also produces very low error (0.028) across the $L_{1}$, $L_{2}$, and $L_{\infty }$ norms. These attacks also transfer well across different models and tasks. The study in [14] shows that both single-step and iterative attacks generalize across fault classification, anomaly detection, and soft sensing. In audio diagnostics, the work in [62] shows the drop in accuracy from 80–90% to a 100% attack success rate. It has been observed that many studies also combine gradient-based attacks with adversarial training or GAN-based augmentation [15,51]. These attacks target a wide range of models, including Gradient Boosting, decision trees, LSTMs, CNNs, and TCNs. Resulting degradation ranges from moderate (15–25%) to nearly complete failure (ASR ≈ 100%).

The performance figures reported in Table 6 are drawn directly from author-reported results under the experimental conditions of each study. The near-complete failure cases reported in [45] (98.7% to 0.03%) and the high attack success rates in [43,53] (89% ASR) originate from white-box settings where the attacker has full access to model gradients. These figures represent a theoretical upper bound on vulnerability under idealized attacker knowledge and should not be interpreted as predictive of attack effectiveness against IIoT systems where gradient access is restricted. Furthermore, results across [46,53,61] are not directly comparable, as they involve different models (ID3, LSTM, deep neural networks), different datasets, and different perturbation budgets.

### 4.2. GAN-Based Generative Methods

Table 7 shows a performance comparison of various GAN-based generative methods. The Temporal GAN framework [57] achieves an AUC of 0.99 and an EER between 0.06 and 0.10. It also runs more than 5.5× faster than MAD-GAN. GANs also exploit dataset imbalances effectively. DB-CGAN [60] hits F1-scores of 99.52% on UNSW-NB15 and 91.32% on NSL-KDD with very low FAR (2.11–2.32%), proving they can create high-quality synthetic minority-class samples to reduce distribution bias and improve imbalanced IDS classification.

At the network level, PS-WGAN [56] generates packet traffic that keeps malicious intent while evading detectors, achieving up to 99% evasion and 30% transferability across CNN, LSTM, GRU (Gated Recurrent Unit), and Kitsune models. This makes them particularly dangerous in black-box scenarios. GANs also simulate zero-day threats well. The study reported in [55] reaches 99.5% detection accuracy on pure zero-day GAN samples, but performance drops in mixed attacks, showing these samples create genuinely challenging distributions. For routing protocols, the study in [47] uses GANs to generate realistic RPL (Routing Protocol for Low-Power and Lossy Networks) attack patterns (sinkhole, Sybil, selective forwarding) that outperform SVM (Support Vector Machine) baselines in classification.

Overall, Table 7 reveals evasion up to 99%, 30% transferability, AUC to 0.99, and F1 up to 99.52% with low FAR (2.11%). Unlike gradient-based attacks that stay within norm bounds, GANs create distribution-realistic samples that are harder to detect—but they need more compute, complex training, and struggle with encrypted traffic or mixed attacks.

The threat-model assumptions in Table 7 are mostly white-box, with two important exceptions. Studies [47,55,60] all rely on white-box access: the GAN-assisted scoring with STM/LTM method in [55], the SMOTE–WGAN augmentation in [60], and the GAN-assisted SVM approach in [47] each require access to the target model or its training data to build and tune their generative components. However, because the study in [57] does not clearly specify its own threat model, it is marked as not applicable (N/A) in this review rather than being assigned a threat-model label. The study in [56] is the main exception, operating in a gray-box-to-black-box regime. Its PS-WGAN framework uses a surrogate classifier to generate adversarial traffic instead of directly targeting the deployed IDS, and the reported 30% transferability across CNN, LSTM, GRU, and Kitsune models shows that the attack remains partially effective without direct access to the target model. This makes [56] the most operationally realistic study in Table 7, as its 99% evasion rate is achieved without assuming white-box access to the detector.

By contrast, the AUC of 0.99 and EER of 0.06–0.10 in [57], the F1-scores of 99.52% (UNSW-NB15) and 91.32% (NSL-KDD) in [60], and the performance gains over standard SVMs reported in [47] should all be read with the understanding that white-box access to model internals or training data was used to guide the generative process.

### 4.3. Poisoning Attacks

The studies in Table 8 show that poisoning attacks target the training phase of industrial ML systems. They distort decision boundaries and cause major performance drops. Unlike evasion attacks that only affect inference, poisoning creates long-lasting and transferable errors. The work in [59] injects DCGAN-generated samples into the training set. As a result, the accuracy decreases from 0.998 to 0.635. After the filtering operation, accuracy only recovers to 0.967. Interpolation and back-gradient poisoning also disrupt different models. For example, the work in [44] achieves success rates of 20–60% across RNN, CNN, and autoencoder-based detectors. After applying gradient smoothing, the recovery is 10–20%. Federated learning causes additional risks because training is decentralized [58]. Here, 88.9% accuracy is achieved under 20% Byzantine clients. Convergence also improves from 42 to 34 rounds while the reduction in communication cost is 35%.

The results in Table 8 assume that the attacker can inject samples into the training pipeline, but how realistic this is depends heavily on the deployment setting. The accuracy drop from 0.998 to 0.635 reported in [59] reflects a centralized training setup with direct access to the dataset, whereas the 88.9% accuracy under 20% Byzantine clients in [58] corresponds to a federated learning scenario where poisoning occurs through malicious model updates rather than direct data access. These are fundamentally different attacker capabilities and should not be interpreted as comparable points on a single severity scale. Likewise, the recovery figures reported after applying filtering or gradient-smoothing defenses ([59], recovery to 0.967; [44], 10–20% recovery) are tied to the specific techniques and settings in those studies and cannot be assumed to generalize to other poisoning scenarios without further evaluation.

### 4.4. Reinforcement Learning Policy Generators

The studies in Table 9 show that RL attacks bring a new level of adaptability to adversarial threats in IIoT and CPS environments. Unlike gradient-based or GAN methods, RL attacks learn policies through sequential decision-making and system feedback. This allows them to dynamically adjust to model responses and operational constraints. Consequently, these attacks can completely break detection systems.

The work in [63] presents an A2C (Advantage Actor-Critic)-based attack that drops IDS detection from 100% to 0% after about 600 training episodes. Similarly, the work in [52] demonstrates a PPO (Proximal Policy Optimization)-based attacker which outperforms static anomaly detection with an F1-score of 0.825 (+14.7% over VAE (Variational Autoencoder) at 0.724 and OmniAnomaly at 0.676). It shows how RL adversaries continuously refine their strategies during interaction. However, this requires heavy computational training and careful policy tuning. At the control level, the work in [17] shows RL attackers using inverse reinforcement learning to exploit deep Q-network controller reward functions. It degrades system behavior and exposes vulnerabilities in high-performance RL-based IIoT systems. It can be observed from Table 9 that RL attacks are the most adaptively powerful threat category, as they deliver complete detection failure and strong anomaly detection degradation. They use environment-aware, sequential optimization. However, these attacks require high computational power and long training (about 600 episodes). These constraints reduce real-world practicality.

It is important to note that the threat-model assumptions behind Table 9 differ markedly across the three studies and are essential for interpreting their reported effectiveness. The study in [63] adopts a black-box setting in which the A2C agent learns a sensor-spoofing policy solely from the IDS’s detection feedback, without access to model internals. This makes the observed drop in detection rate from 100% to 0% arguably the most operationally realistic result in this category, but it still relies on the attacker having long-term, repeated interaction with the system over roughly 600 episodes. By contrast, the study in [17,52] assume gray-box adversaries. The PPO-based attacker–defender framework in [52] requires knowledge of the multivariate time-series structure and access to intermediate signals to shape its adaptive perturbation policy. The inverse-reinforcement learning approach in [17] similarly relies on partial knowledge of the deep Q-network controller’s reward structure in order to approximate and exploit it. As a result, the F1-score improvements reported in [52] and the vulnerability illustrated in [17] rest on attackers with more system insight than in a pure black-box setting, but less than the full access assumed in typical gradient-based white-box attacks (Section 4.1).

Taken together, these studies show that RL-based attacks span a broader spectrum of threat-model realism than any other category in this review—from the black-box, feedback-only scenario in [63] to the gray-box, system-aware settings in [17,52]. This range should be taken into account when evaluating their practical threat level for real IIoT deployments.

### 4.5. Saliency-Based Attacks

The studies in (Table 10) show that saliency-based attacks employ feature-importance information to produce precise perturbations. Consequently, they target only the most influential features. This makes the manipulation more localized and often more stealthy. The work in [48] shows that JSMA reduces Random Forest accuracy by about 6 percentage points and J48 accuracy by 11 points. For time-series IIoT data, the work in [49] shows that models normally achieve around 95% accuracy but become unstable under targeted saliency attacks. In malware classification, the work in [54] finds that Jacobian-based attacks increase accuracy by about 6% and reach an AUC of 0.958. However, when adversarial samples are more than 65% of the training data, overfitting is observed. The work in [62] shows CNNs with 80–90% accuracy. However, JSMA achieves 100% attack success for specific classes. It can be concluded that saliency-based methods provide precise and stealthy manipulation as compared with gradient-based attacks. Nevertheless, they require access to feature-importance information. Moreover, high-dimensional or encrypted data are additional constraints in this case.

The threat-model assumptions in Table 10 span all three categories, which is noteworthy given that saliency-based attacks are often treated as purely white-box. The studies reported in [49,54] both use white-box settings: the JSMA-based perturbations on sensor and actuator data in [49], and the Jacobian-based attack on the KBL model in [54], each require direct access to model gradients to compute the saliency maps that guide perturbations. Their reported results—95% accuracy in [49] and a 6% accuracy gain with an AUC of 0.958 in [54] (with overfitting once adversarial samples exceed 65% of the training data)—therefore assume full attacker access to model internals. The study in [48] operates in a gray-box regime. Its JSMA attacks on RF and J48 ICS IDS models rely on feature-importance information rather than full gradient access, making the observed 6-point (RF) and 11-point (J48) accuracy drops more realistic for an adversary with partial but not complete system knowledge. The study in [62] is the only black-box study in this group: although it applies gradient-based JSM-family attacks (FGSM, PGD, C) to CNN spectrograms, the reported 100% attack success for specific classes is achieved under black-box conditions, indicating that saliency-driven perturbations crafted on a surrogate model can transfer effectively to the target without direct access.

Taken together, this range—from the black-box transferability in [62] through the gray-box setting in [42] to the white-box requirements in [49,54]—shows that saliency-based attacks do not inherently imply a white-box threat model. Their reported effectiveness needs to be interpreted in light of each study’s specific access assumptions.

### 4.6. False Data Injection Attacks

Although Table 11 includes only one study, it identifies a distinct adversarial threat in cyber–physical systems (FDIA). It’s important to distinguish FDIA from poisoning attacks (discussed in Section 4.3). Poisoning tampers with the training data by inserting malicious samples, while FDIA targets the inference or online monitoring stage by altering live sensor values or process variables. Unlike poisoning, FDIA must still respect the system’s physical dynamics and invariants to avoid detection. The two overlap only when FDIA-tainted sensor data is later used for retraining, creating a cross-phase attack path that deserves further study. In [16], a dual-VAE framework with GRU-based temporal encoding generates realistic FDIA samples. It uses a min–max game between generative and discriminative models to learn time-dependent patterns in smart grid data. It achieves higher AUC-ROC and AUC-PR than GRU, VAE, and other adversarial baselines. However, its architecture and training introduce significant computational overhead. This may limit real-time deployment in large-scale power systems.

Table 11 includes only one study, so it offers limited evidence for this attack category. The study reported in [16] assumes a gray-box threat model: the dual-VAE with GRU-based temporal encoding does not need access to the internals of the detection model, but it does require knowledge of the smart grid’s data distribution and temporal dynamics to generate realistic FDIA samples. The study reports better AUC-ROC and AUC-PR than GRU, VAE, and other adversarial baselines, but only in relative terms—absolute values are not provided in the information available to this review. It also explicitly notes significant computational overhead, which may hinder real-time use in large-scale power systems.

Overall, the gray-box assumption in [16] is broadly in line with typical FDIA scenarios, where an attacker needs process-level knowledge to inject plausible false sensor data rather than direct access to the ML model. However, because this category is based on a single study, these conclusions should be seen as indicative, not definitive, for FDIA in IIoT.

### 4.7. Hybrid-Generated Attacks

The studies in Table 12 show that hybrid-generated attacks combine multiple perturbation strategies. Consequently, these attacks become more diverse and more effective than single-method approaches. To avoid this category becoming a catch-all, a study is classified as hybrid only when it combines two or more of the six other mechanism-defined categories within a single attack pipeline. The work in [50] integrates FGSM and PGD to increase attack success by 36% and reduces F1-score by 61%. The PCAA method in [65] drops RF-based TSA accuracy from 0.98 to 0.38 (about 60 percentage points). Recall falls from 1.0 to 0.40, and F1 decreases from 0.99 to 0.55. Even in black-box transfer, it still affects DNN (0.75 accuracy), KNN (0.65), and SVM (0.85). Similarly, the study in [64] uses temporal augmentation—noise to increase accuracy up to 96.83% and F1 above 94%. In short, Table 12 identifies a higher amount of degradation by combining multiple techniques. However, their complexity increases computational cost and implementation difficulty. The work in [66], combines optimization-based adversarial generation with a GAN-based attack strategy. The study targets ML-based IDSs in ICS under a black-box evasion setting. Unlike generic adversarial attacks, the generated samples are constrained by ICS protocol validity, legal feature ranges, and payload preservation, so the adversarial packets can evade detection while still maintaining their malicious effect. The proposed attacks are evaluated against injection, function-code, and reconnaissance attacks on a semi-physical ICS testbed. The results show that the Optimal Solution Attack achieves an ASR of 20.958% for injection attacks, 81.201% for function-code attacks, and 100% for reconnaissance attacks. The GAN-based attack also reaches 100% evasion on reconnaissance attacks and provides a faster generation process. These findings show that hybrid adversarial generation can expose practical weaknesses in ICS IDSs, especially when the attack design considers both ML evasion and industrial protocol constraints.

The threat-model assumptions in Table 12 are mixed and, in the case of [65], even cover both extremes within a single study. This study [50] adopts a black-box setting in which the combined FGSM–PGD pipeline is applied without internal access to the X-IIoTID-based IDS, so its reported 36% increase in attack success and 61% drop in F1-score are directly relevant to externally positioned adversaries. Similarly, the study in [66] evaluates black-box adversarial evasion against an ML-based IDS in ICS, but with stronger attention to industrial constraints, since the generated samples must preserve protocol validity, legal feature ranges, and the malicious payload. In contrast, the study reported in [64] assumes a white-box setting: its temporal perturbation and augmentation strategy for the LSTM Autoencoder requires access to the model to guide the augmentation process, which is important context for interpreting its reported 96.83% accuracy and F1-scores above 94%.

The work in [65] is distinctive because it reports results under both white-box and black-box conditions. In the white-box case, the PCAA method uses gradient-based optimization against the RF-based TSA model, reducing its accuracy from 0.98 to 0.38. The same study then evaluates black-box transfer, reporting degraded accuracies for DNN (0.75), KNN (0.65), and SVM (0.85), showing that the attack retains partial effectiveness even without direct access to these models. The study in [66] adds another black-box perspective by combining optimal-solution and GAN-based generation, where the strongest reported evasion appears in reconnaissance attacks, reaching 100% ASR. This makes [65] the only study in this group to provide a within-study comparison of white-box and black-box effectiveness. while the study in [66] shows how black-box hybrid attacks can remain practical when ICS protocol and payload constraints are explicitly considered. These results suggest that hybrid attacks incorporating gradient-based components do not need white-box access to every target model to remain partially effective, although their strongest impacts still rely on white-box access to at least one model in the pipeline.

### 4.8. Threat-Model Assumptions and Real-World Applicability

A cross-category analysis of the reviewed studies shows that white-box assumptions are still the most common way of evaluating adversarial attacks in IIoT. As summarized across Table 6, Table 7, Table 8, Table 9, Table 10, Table 11 and Table 12.

Across the 28 reviewed attack studies, 50% rely exclusively on white-box assumptions, with most of these concentrated in gradient-based attacks (6 of 9 studies) and several saliency- and GAN-based approaches. In contrast, only 21% of studies evaluate attacks under purely black-box conditions, while 18% adopt gray-box assumptions. Three studies [14,56,65] examine more than one threat-model setting within the same work, and only the study in [65] provides a direct comparison between white-box and black-box attacks against the same target model. This distribution highlights a significant imbalance in the current literature. Although adversarial machine learning research in IIoT continues to expand, half of the reviewed studies still depend on idealized attacker-knowledge assumptions. Meanwhile, black-box evaluation—which is arguably the most realistic scenario in operational IIoT environments, where attackers rarely have access to gradients, model architectures, or loss functions—remains relatively uncommon.

Gradient-based attacks Table 6 and saliency-based attacks Table 10 predominantly assume strong model access, including gradient availability, architecture knowledge, loss-function transparency, or saliency/Jacobian information. These settings are useful for stress-testing IDS robustness, but they should be interpreted as theoretical upper bounds on adversarial vulnerability rather than direct evidence of operational exploitability in deployed IIoT systems, where such internal access is rarely achievable without insider privileges, model leakage, or physical compromise.

GAN-based methods Table 7 show a more mixed profile. Some studies rely on access to the training distribution, surrogate models, or generated samples, while others still involve stronger white-box assumptions. Therefore, their realism depends on whether the adversary is assumed to access the target model, only approximate the data distribution, or transfer attacks from a substitute model. RL-based attacks Table 9 generally adopt more deployment-relevant assumptions than gradient-based attacks, since they often rely on interaction with the environment, reward feedback, or observable system behavior rather than direct gradient access. However, several RL-based settings remain gray-box because they require partial knowledge of the control process, system dynamics, or training environment.

FDIA studies Table 11 are best interpreted as attacks assuming a process-aware gray-box threat model because their feasibility depends on knowledge of system topology, sensor relationships, and physical invariants rather than access to ML model internals. Poisoning attacks Table 8 present a context-dependent realism profile: they are credible in federated, online, or continual-learning deployments where adversaries may influence the training pipeline, but require stronger justification in centralized offline settings. Hybrid attacks Table 12 inherit the strongest access assumption from their constituent methods and should therefore be interpreted according to the most demanding component. Overall, the reviewed literature shows that many reported attack results are produced under strong access assumptions, while fewer studies evaluate adversarial robustness under access constraints that closely match realistic IIoT deployment environments.

A further point that emerges from the threat-model and dataset classifications added in (Section 4) is that the heterogeneity across studies is not random; it is largely driven by the attack mechanisms themselves. Threat-model assumptions tend to cluster by attack type in ways that match each mechanism’s basic requirements: gradient-based attacks (Section 4.1) are uniformly white-box because access to gradients is built into how they work, whereas saliency-based attacks (Section 4.5) appear under white-, gray-, and black-box settings because feature-importance information can be derived with different levels of model access, depending on the study.

A similar pattern appears for datasets. Modality clusters by attack category in line with where each mechanism naturally operates: FDIA (Section 4.6) is applied only to sensor and process data because it is defined around manipulating physical measurements, while gradient-based attacks span image, audio, hydraulic, and ICS data because the underlying optimization is largely modality-agnostic.

This perspective re-frames the cross-study variability discussed in (Section 8.1). Much of the apparent heterogeneity reflects systematic differences in what each mechanism requires and where it can be applied, rather than inconsistent experimental practice alone. A practical implication is that future standardization efforts (Section 7.2) may be more effective if they focus on mechanism-specific clusters (for example, standardizing FDIA evaluation around process-constrained sensor benchmarks) instead of relying on a single benchmark suite meant to cover all seven attack categories in the same way.

Based on the domain-level clustering developed in Section 4.9, a minimally realistic threat model also differs by IIoT application. For anomaly and intrusion detection systems (SWaT/WADI-type deployments), a credible minimum is gray-box access to network traffic and detector outputs without direct model gradients, since deployed IDS models are rarely exposed to attackers. For process control and state estimation (smart-grid and FDIA settings), the minimum realistic assumption is process-aware gray-box access—knowledge of sensor-actuator relationships and physical invariants rather than ML model internals—consistent with the FDIA discussion in Section 4.6. For fault diagnosis and condition monitoring, physically bounded black-box or gray-box access is appropriate, since perturbations must respect sensor measurement ranges. For RL-controlled closed-loop systems, a minimum realistic model requires only interaction with system feedback, as demonstrated in [63], rather than access to the underlying reward function or policy network. Studies assuming full white-box access in these settings should explicitly flag this as a theoretical upper bound on vulnerability rather than a deployment-representative result.

### 4.9. Observations by IIoT Application Domain

While the earlier sections organize the literature by attack mechanism, the studies also naturally group into specific IIoT application domains, and looking at these clusters reveals where particular attacks and defenses are most often used.

**Water treatment and process control (ICS/CPS).** Studies using SWaT and WADI [49,52,57,64] mainly evaluate saliency-based attacks (JSMA, [49]), GAN-based reconstruction ([57]), RL-based approaches (RAAD/PPO, [52]), and hybrid temporal augmentation [64]. In this domain, GAN-based reconstruction detection and RL-based adaptive detection report the strongest performance (AUC 0.92–0.99 and F1 = 0.825. This likely reflects how well these process-control datasets capture temporal and sequential dependencies, which both GAN-reconstruction and RL-based methods are designed to exploit. By contrast, the saliency-based result on SWaT-related ICS data reports only accuracy, without comparable degradation or recovery metrics, which limits direct comparison. A related but physically grounded line of work targets a semi-physical S7comm water-tank testbed rather than SWaT/WADI [66], where Opt. attack and GAN-based evasion attacks are generated against an ensemble (stacking) IDS and validated through real physical effects—water level surges, PLC shutdown, and packet exfiltration—rather than simulated process variables alone. This testbed-level validation, also extended to Modbus gas-pipeline and IEC 104 honeypot data, complements the SWaT/WADI cluster by showing that GAN- and optimization-based evasion generalize beyond simulated benchmarks to real controller hardware, though at the cost of dataset scale and protocol diversity compared with SWaT/WADI.

**Smart grid and power systems.** Work in this domain [16,61,65,71,74] is dominated by FDIA (the only FDIA study, [16], targets smart grid data) and gradient-based attacks on grid-monitoring models [61,65,74]. On the defense side, adversarial training with defensive distillation [61] raises SIEM accuracy from 30–40% to above 80% under attack, and the CWT-CNN method with Gaussian noise augmentation [74] maintains 94.06% accuracy under combined FDI and FGSM attacks. The focus on FDIA and physically constrained gradient attacks aligns with the role of physical invariants in power system state estimation, where both attacks and defenses must respect process-level constraints (e.g., power-flow equations) that are less central in purely network-traffic settings.

**Industrial network intrusion detection (IIoT/NIDS).** The largest cluster [50,55,56,60,68,69,84,85,86] uses network-traffic datasets such as X-IIoTID, Edge-IIoTset, NSL-KDD, UNSW-NB15, and NF-BoT-IoT/NF-ToN-IoT. This domain shows the widest variety of attack types, including GAN-based traffic generation ([56], 99% evasion), hybrid FGSM+PGD ([50], 36% increase in attack success), and gray-box transfer attacks. On defense, GAN-based and ensemble methods report the highest absolute performance (e.g., [83]: >99% accuracy and F1; [84]: 99.21% accuracy). However, this performance can drop sharply under mixed-attack conditions ([55]: F1 from 98.09% to 43.6% under GAN-Theft attacks). This suggests that, although NIDS-focused defenses achieve some of the strongest headline numbers, they may also be particularly sensitive to attack diversity.

**Manufacturing, fault diagnosis, and condition monitoring.** Studies using TEP, WM-811K, and hydraulic system datasets [14,15] concentrate on gradient-based attacks and adversarial training. The study in [15] reports 10–15% robust-accuracy gains from PGD-based adversarial training on RTCN/TCN models, indicating that adversarial training is relatively well matched to the continuous, sensor-driven time series typical of this domain, where perturbations are more tightly constrained by physical sensor ranges than in image or network-traffic data.

**Federated and distributed IIoT.** Federated-learning studies [58,73,75,80,87] focus mainly on poisoning attacks (e.g., Byzantine poisoning in [58]) and hybrid or layered defenses that combine robust aggregation with anomaly detection or privacy-preserving techniques. These works report accuracy gains in the 3.7–6.8% range, along with improvements in convergence and communication efficiency, but they also consistently highlight higher training time, encryption overhead, or assumptions such as static network topology as limiting factors [73,87]. In this domain, defense effectiveness is more often reported together with system-level costs (communication, convergence, latency) than in centralized settings, reflecting the inherently distributed nature of the threat model.

Finally, these domain-level observations are based on how studies cluster by dataset and application context within this review’s corpus, not on controlled cross-domain comparisons inside any single study. None of the reviewed work evaluates the same attack or defense method across multiple IIoT domains under a consistent protocol, which is itself a gap highlighted in Section 7.1.

#### Cross-Domain Suitability Synthesis

Drawing the five domain clusters discussed above together, clear patterns emerge regarding which methodologies are best suited to which IIoT application context, and why. In water treatment and process control, GAN-based reconstruction and RL-based adaptive detection perform best (AUC 0.92–0.99, F1 = 0.825) because these datasets capture strong temporal and sequential dependencies that both mechanisms are explicitly designed to exploit; static gradient-based attacks are comparatively less informative here, since they do not model the closed-loop, time-dependent nature of the process. In smart grid and power systems, FDIA and physically constrained gradient attacks dominate because both attacks and their defenses must respect power-flow equations and other physical invariants, giving process-aware methods a natural advantage over generic feature-space attacks. In industrial network intrusion detection, GAN-based and ensemble ML defenses report the strongest absolute performance, but this is also the domain where performance is most sensitive to attack diversity, suggesting that method selection here should weigh robustness under mixed attacks as heavily as peak accuracy. In manufacturing, fault diagnosis, and condition monitoring, adversarial training is comparatively well matched, since sensor-driven time-series data is tightly bounded by physical measurement ranges, constraining the perturbation search space in a way that favors training-based robustness over post hoc detection. In federated and distributed IIoT, hybrid and layered defenses are preferred by necessity, since poisoning in this setting occurs through malicious model updates rather than direct data access, requiring detection and aggregation-level defenses to work together rather than in isolation.

Overall, methodology suitability across this review’s corpus tracks two properties of the target domain: the extent to which system behavior is temporally or sequentially structured, which favors GAN- and RL-based methods, and the extent to which perturbations must respect physical or process-level constraints, which favors FDIA and physics-aware gradient methods. Domains lacking either property, such as generic network-traffic detection, show the least methodological convergence and the widest variation in reported performance, which is consistent with the sensitivity to attack diversity noted above for the NIDS cluster.

## 5. Defense Mechanisms and Detection Frameworks for Adversarial Attacks in IIoT

The analysis of the selected articles shows a wide range of defense methods against AML threats. The following subsections provide a comprehensive performance comparison of selected articles in each defense method category.

### 5.1. Adversarial Training Defense Mechanism

Across the reviewed literature in Table 13, adversarial training appears as one of the most widely used defense strategies. Despite differences in models, attack types, and application domains, a consistent pattern emerges. Models retrained with adversarial examples generally recover their performance and often become more resilient to gradient-based attacks.

Across the studies in Table 13, adversarial training appears as a reactive way to improve robustness. Models are retrained, either fully or selectively, using adversarial or attack-like samples. This helps them recover detection or classification performance under evasion and poisoning attacks. The results show that the method works, but the level of improvement varies by model type, attack strength, and application domain. In classical machine learning models, the gains are small. Retraining RF and J48 on JSMA samples [48] recovers only about 6% for RF and 11% for J48. These shallow models benefit, but only slightly, and the evaluation covers only a single-step attack, not stronger perturbations.

In deep IDS and IIoT classifiers, the improvements are larger but still limited. A DNN-based IDS trained with adversarial learning [51] increases accuracy from 93.7% to 97.9% and raises F1 by about 4% under FGSM and PGD with ϵ≤0.15. Noise-augmented training with CWT features [74] keeps accuracy at 92.08% under moderate noise, but performance drops to 84–86% when the perturbation grows to |ϵ|=0.2. These results show that robustness gains weaken as attacks become stronger. For temporal and industrial monitoring tasks, PGD-based adversarial training with RTCN/TCN models [15] improves robust accuracy by about 10–15% across several attacks. This is a strong gain, but it requires expensive PGD training and still focuses on norm-bounded threats. In EIFDAA [68], models are very weak before retraining, and therefore the recovery is limited.

For poisoning attacks, robust regression-based training in [71] reduces undetected attacks by about 30% and increases F1 by up to 61%. However, it also raises false alarms. Selective retraining in [54] improves accuracy from 71.0% to 86.08%, and AUC rises to 0.958. Ensemble adversarial training in [69] achieves accuracy above 99% with ADR below 0.15%. These are the strongest results, but they require complex models and more resources. AT-GVAE [16] also improves AUC-ROC and AUC-PR and supports FDIA localization, but its gains are reported relative to baselines. Overall, Table 13 shows that adversarial training can raise robustness from small gains of 4–6% in standard IDS models to 10–15% in deep and temporal models.

### 5.2. Run-Time Monitoring Anomaly Detection

Table 14 provides the results of [78,87,88], which fall in the Run-time Monitoring Anomaly Detection category. In [78], the authors use a GAT-based APT detection model to learn both sensor behavior and the topology among nodes through graph attention and masked self-attention. The model gives strong results, reaching 96.97% accuracy on DAPT2020 and 95.97% on Edge IIoT. Even so, the prediction time is relatively high, at about 20–21 s, which makes the approach more suitable for offline analysis or batch-based monitoring rather than time-critical detection.

The study in [87] looks at the problem from a federated IIoT perspective. Its framework combines adaptive anomaly detection with homomorphic encryption and differential privacy, so the focus is not only on detection performance but also on privacy and secure collaboration between IIoT nodes. The reported results show an F1-score of 88.5%, an FPR of 2.7%, and an adversarial success rate reduced to 8.3%. The framework also reduces communication overhead by 53% and improves convergence speed by 23%. However, these improvements are not without cost. The encryption process adds computational overhead, and the assumption of a static network topology may not fully reflect more dynamic IIoT deployments.

The work in [88] is different because it focuses directly on adversarially robust runtime detection in an ICS setting. The authors propose RADIANT, a reactive autoencoder-based IDS that avoids adversarial retraining. Instead of training the model on specific adversarial samples, RADIANT uses three autoencoders: a denoising autoencoder, a benign-only autoencoder, and an attack-only autoencoder. The extracted latent features and reconstruction errors are then used to support the final classification decision. Against gray-box targeted evasion attacks, RADIANT achieves an F1-score of 91.4% and an FPR of 1.2% under HopSkipJump, while under ZOO it achieves an F1-score of 85.9% and an FPR of 1.4%. These results suggest that reconstruction-error-based runtime monitoring can make IDS models more resistant to adversarial inputs while keeping false alarms low.

### 5.3. Hybrid/Layered Defenses

These methods combine several protection layers to overcome the limits of single defenses. A major trend is fault-tolerant federated learning with multiple protection layers. In [58], robust aggregation, anomaly detection, blockchain consensus, and continual updates are integrated to defend against poisoning and gradient manipulation. This improves accuracy from 85.2% to 91.7% on MNIST and from 61.5% to 68.3% on CIFAR-10. The system also keeps 88.9% accuracy with 20% Byzantine clients and converges 19% faster. The work in [73] presents another hybrid method. It mixes federated learning with GAN-generated synthetic data and adaptive aggregation. It provides 96.8% accuracy and up to 95.1% F1-score. Therefore, a 6.8% accuracy gain is achieved with 34% faster convergence and 42% fewer communication rounds. Nevertheless, it increases training time by 29% and raises communication costs.

Adversarial training with defensive distillation for LSTM-based SIEM models is presented in [61]. It recovers accuracy from 30–40% under attack to above 80%. Similarly, game-theoretic defenses in CPS [72] increase time-to-breach by 25%. Privacy-focused hybrids combine cryptography with learning. A multi-layer security framework [77] achieves low latency (re-encryption <250ms, homomorphic encryption <60ms) and small communication cost (≤20 kB) for 20 nodes). A blockchain-based FL system [80] improves accuracy by 3.7%, reduces latency by 28%, and lowers energy use by 21%. The bio-inspired V1 front-end [46] yields its strongest robustness gain on the industrial Conveyor-V3 dataset under PGD-20. Specifically, the baseline ResNet-50 accuracy collapses to (0%) by (ϵ = 0.01), whereas V1+ResNet-50 retains approximately (50%) accuracy under the same perturbation level and does not fully degrade until around (ϵ≈ 0.04). The CIFAR-10 results under PGD-5 show the same directional effect, but with a smaller margin of approximately (+5)–(9) percentage points across (ϵ = 0.05)–(0.15). This indicates that the V1-based architectural defense generalizes beyond the industrial dataset, while delivering its largest practical benefit in the industrial visual-monitoring setting rather than in an IIoT-specific cybersecurity scenario.

Overall, Table 15 shows accuracy gains of 3.7–6.8% (and up to 40–50 points in recovery cases), faster convergence (34%), and lower communication (42%). But these benefits come with higher training time, more overhead, and greater system complexity. Hybrid defenses offer strong protection, but scaling them to large IIoT deployments remains challenging.

### 5.4. Detection-Based Defense

The studies in Table 16 show that detection-based defenses work by identifying adversarial, poisoned, or abnormal inputs at inference time. They do not retrain the model but instead monitor inputs for suspicious patterns. These methods achieve strong accuracy, F1-score, AUC, and ASR reduction, although results vary with attack type and data quality.

Hybrid generative–discriminative detectors perform especially well. STRIP with CycleGAN [67] reduces ASR from 98–100% to 0–3% with only a small drop in clean accuracy. LSTM–GAN reconstruction [57] reaches AUC values of 0.92–0.99 and EER of 0.06–0.10, and runs 5.5× faster than MAD-GAN. These methods detect even zero-day attacks, but require heavy GAN training. Ensemble and multi-view detectors add stability. An optimized SVM ensemble in [82] achieves 91% accuracy, 0.94 precision, and 0.95 recall for malware evasion. GAN-SVM routing detection [47] also outperforms standard SVMs for RPL attacks. RAAD [52] improves F1-score by 14.7% (0.825 vs. 0.724 for VAE). SAMKNN [55] reaches 98.09% accuracy on NF-BoT-IoT but drops to 43.6% under GAN-Theft attacks. MARD [70] performs best, with 99.56% accuracy on ICU data across PGD, BIM, and other attacks, though it weakens on low-dimensional data.

SHAP-based [79] achieves F1-scores of 0.94–0.99 while reducing training time by 42–90%. Trust-based methods [75,76] remain stable within ±1–2% accuracy under moderate risk. Federated IDS approaches [81] reach F1-scores of 0.92–0.98 with 50–75% less communication. Hybrid DRL–GAN [83] achieves over 99% accuracy and F1. GAN variants such as DB-CGAN [60], CCGAN [84], and memory-augmented autoencoders [86] also perform well, with F1 up to 99.5%. Overall, Table 16 shows high performance across many detectors, with accuracy often between 95–99.56%, F1-scores up to 99.26%, and ASR reduced to 0–3%. However, performance drops under mixed attacks or imbalanced data.

### 5.5. Cross-Method Comparison and Synthesis

Table 17 synthesizes the four defense categories analyzed in Section 5.1, Section 5.2, Section 5.3 and Section 5.4 along the dimensions of robustness and effectiveness, computational overhead, and applicability to resource-constrained IIoT edge deployments.

Three insights follow from this comparison that are not visible when each category is examined in isolation. First, there is a near-inverse relationship between robustness and deployability across this literature: the categories with the strongest reported robustness—detection-based and hybrid—are also the most computationally demanding, while the most edge-feasible category, adversarial training once trained, offers only moderate and attack-specific gains. This suggests that no single defense category, as currently studied, satisfies both the robustness and resource constraints of real IIoT deployments simultaneously. Second, computational overhead is unevenly reported across categories: detection-based and hybrid methods are the most likely to report overhead figures (Section 5.3 and Section 5.4), while adversarial-training studies rarely quantify inference-time cost, making a fully consistent overhead comparison across all four categories only partially possible with currently available data. Third, robustness claims across categories are not measured against a common attack set; a detection-based method reporting near-perfect accuracy and a runtime-monitoring method reporting strong false-positive control may not have been evaluated against comparable attack strength, which limits how far the finding that detection-based methods post the highest absolute performance Section 5.4 can be generalized across categories. Together, these observations indicate that future comparative evaluation should report robustness and overhead jointly, under a shared attack budget, rather than as separately optimized figures—a gap this review connects to the standardized reporting protocol proposed in Section 7.2.

## 6. Classification According to the Datasets

Section 4 and Section 5 have provided performance comparisons in terms of various attack types and corresponding defense mechanisms. Nevertheless, a systematic analysis of the selected articles based on their datasets is equally important. The objective is to illustrate the effect of dataset type on AML results in IIoT settings. First, Section 6.1 provides an in-depth discussion on synthetic datasets. They offer controlled environments for testing robustness. However, they also raise concerns about realism and standardization. Subsequently, Section 6.2 presents published datasets. Although they are limited, they capture real operational behavior. Finally, Section 6.3 explains the excluded studies and non-dataset approaches.

### 6.1. Analysis of Synthetic Datasets

Table 18 summarizes the synthetic datasets used in the selected articles. It can be observed that synthetic data plays an important role in AML research for IIoT because collecting real industrial data is difficult. In other words, synthetic datasets are a practical method for validating models in controlled settings.

#### 6.1.1. Generation Techniques

Table 18 divides synthetic datasets into three main types: GAN-based data, gradient-based adversarial data, and simulation-based data. It shows the variety of methods used in the selected studies.

- Examples of GAN-based methods include VOneGAN for industrial images [46], PS-WGAN for adversarial traffic [56], and memory-augmented GANs for abnormal IIoT data [86]. Other variants such as DB-CGAN [60] and CycleGAN [67] generate minority-class samples.
- Examples of Gradient-based adversarial methods include JSMA manipulation [48,49], false data injection for smart grids [16,74], and physics-aware adversarial samples.
- Simulation-based generation includes RPL routing attack simulators [47], DRL control simulations [17], and CPS attack-graph models [72]. Other studies combine synthetic and real ICS data with TEP simulations [44], or simulate federated learning systems under attack [75,80]. Large-scale power system simulations [65], microgrid sensor perturbations [61], and trust-score simulations for IoT devices [76] also fall here.

The wide variation across methods shows the need for more unified benchmarks to support fair comparison in IIoT settings.

#### 6.1.2. Balancing Realism and Control in Synthetic IIoT Data

Synthetic datasets provide strong experimental control. In this category of datasets, researchers can adjust attack type, strength, frequency, and system settings as per their requirements. Therefore, rare or high-impact events that are hard to observe in real environments can also be tested. Adversarial augmentation methods that modify real industrial signals [64] provide a partial solution. They keep the realism of real data while adding the control needed for systematic testing. Several studies [15,48,74] also use hybrid approaches that mix real measurements with synthetic adversarial perturbations. Overall, no single data generation method meets all evaluation needs.

### 6.2. Analysis of Published Datasets

Table 19 summarizes the published datasets most frequently employed in AML research for IIoT.

#### 6.2.1. Dataset Diversity and Application Domains

The analysis of selected articles shows that many works rely on well-known public datasets. Image datasets such as MNIST, CIFAR-10/100, Conveyor-V3, and T-LESS are widely used in industrial vision and IIoT applications. They support research on backdoor defense, adversarial training, and synthetic image generation [46,59,67,80]. Cyber–physical process datasets, especially SWaT and WADI, are the main benchmarks for intrusion and anomaly detection. They enable testing against gradient-based, GAN-based, and reinforcement learning attacks [49,52,57,64]. Network intrusion datasets such as UNSW-NB15, NSL-KDD, CIC-IDS2017, CSE-CIC-IDS2018, Kitsune, CIC-IoT23, and NF-BoT-IoT/NF-ToN-IoT form the core of adversarial NIDS evaluation. They support studies on evasion, poisoning, imbalance correction, zero-day detection, and federated IDS design [52,55,56,60,63,73,84,86]. Industrial and smart-grid datasets, including Pecan Street AMI, Electra ICS, gas pipeline SCADA, water tank systems, the MSU/ORNL power system dataset, and power distribution testbeds, extend adversarial testing to safety-critical settings. A representative example is the self-collected S7comm water-tank dataset from a semi-physical ICS testbed (real Siemens PLC controlling a simulated quadruple-tank process), evaluated alongside the Mississippi State Modbus gas-pipeline dataset and a custom IEC 104 honeypot dataset to test evasion attacks against an ensemble IDS [45,53,66,71,81,88].

Fault and manufacturing datasets such as TEP and WM-811K wafer maps are used to study adversarial robustness and transferability in industrial inspection and fault diagnosis [14]. Large IIoT intrusion datasets like X-IIoTID and Edge-IIoTset provide more realistic IIoT environments. They support multi-attack evaluation, federated detection, and hybrid adversarial testing [50,68,69,78,87]. New IoMT datasets such as CICIoMT2024 and ICU healthcare data extend adversarial analysis to medical systems. They allow testing of multi-protocol attacks and defense methods in sensitive healthcare environments [70]. Specialized datasets, including Android malware corpora, acoustic data, and hydraulic system condition monitoring datasets, broaden adversarial evaluation to mobile security, industrial acoustics, and sensor-based diagnostics [15,54,62]. Synthetic and hybrid datasets such as DS2OS highlight the growing role of generated data in IIoT security. They support controlled testing of anomaly detection and adversarial learning methods [83,85].

#### 6.2.2. Adversarial Use and Limitations

Published datasets are rarely used in their original form. Most studies modify them with adversarial methods such as gradient-based attacks, GAN-generated samples, reinforcement learning-based anomalies, or feature-level manipulation. Similarly, in [66], the Modbus gas-pipeline and IEC 104 datasets are not used in their original form but are combined with newly generated adversarial network packets—produced via Opt. attack and GAN-based attack algorithms to test evasion against an ensemble (stacking) IDS in ICSc, with attack effects physically validated through water level changes and PLC state transitions. SWaT and NSL-KDD are often used to test how IDS models fail and recover under different adversarial settings [49,52,57,64,84,86]. Datasets such as X-IIoTID, Edge-IIoTset, and UNSW-NB15 are used to study multi-attack robustness, federated learning scenarios, and hybrid adversarial frameworks [50,60,68,69,73,78,87]. Recent work also extends these evaluations to IoMT systems. Datasets like CICIoMT2024 and ICU healthcare data are used to test defenses against multi-protocol and medical-specific attacks [70].

Overall, Table 19 shows that published datasets serve mainly as a starting point. Researchers adapt and extend them to explore a wider range of adversarial behaviors that the original datasets do not cover.

### 6.3. Excluded Studies and Non-Dataset Approaches

In addition to the published datasets summarized in Table 19, several studies were reviewed that do not rely on dataset-driven evaluation and were therefore excluded from the dataset analysis. For instance, the study in [77] provides conceptual grounding and threat-modeling insights for AML in IIoT but does not enable empirical robustness testing or comparative evaluation using datasets. Similarly, this study in [58] evaluates FL framework with MNIST and CIFAR-10 image datasets. Nevertheless, the experimental evaluation does not involve IIoT-related datasets. In addition, the study in [51] investigates real-world adversarial attacks on process control systems that rely on inertial sensors and actuators. This study demonstrates the effect of adversarial manipulation on physical processes in realistic settings. However, it does not employ published benchmark datasets or generate synthetic datasets. Therefore, it is excluded from the dataset categories.

### 6.4. Diagnostic Assessment and Benchmark Recommendations

Beyond identifying which datasets were used, an important question for the field is whether existing benchmarks adequately reflect the operational constraints of IIoT environments. These constraints include real-time control behavior, noisy or drifting sensor measurements, resource-limited devices, safety-critical response requirements, and physical process invariants. Based on the evidence summarized in Table 18 and Table 19, this section provides a diagnostic assessment of the most commonly used benchmarks and offers recommendations for future adversarial ML evaluation in IIoT.

SWaT and WADI remain among the most operationally grounded benchmarks for adversarial evaluation in industrial cyber–physical systems. Both datasets originate from instrumented physical testbeds and contain realistic sensor-actuator relationships, temporal dependencies, and process dynamics. This makes them suitable for evaluating attacks and defenses that must preserve physical consistency rather than only modify abstract feature vectors. However, their coverage of network-layer adversarial behavior is limited, and they do not fully capture protocol-level manipulation, heterogeneous IIoT device profiles, or edge-resource constraints.

In contrast, NSL-KDD and UNSW-NB15 are widely used but less representative of IIoT deployment conditions. These datasets were originally designed for general network intrusion detection rather than industrial control or IIoT environments. As a result, they do not adequately reflect deterministic control-loop timing, sensor-actuator coupling, constrained edge devices, or safety-critical operational requirements. Their continued dominance in the reviewed literature therefore indicates a benchmark realism gap: many studies evaluate adversarial robustness using convenient legacy NIDS datasets rather than datasets that reflect the operational structure of IIoT systems.

More recent datasets such as X-IIoTID and Edge-IIoTset represent a more IIoT-aligned generation of network-security benchmarks. They include broader IIoT device profiles, modern attack types, and more realistic network behavior than legacy NIDS datasets. For this reason, they should be prioritized in future adversarial evaluation studies, especially when the objective is to evaluate network-layer attacks, intrusion detection, or edge-oriented IIoT security. However, they should ideally be complemented with physical process datasets when the attack claims involve control behavior, process consistency, or safety impact. For smart-grid and broader CPS adversarial evaluation, datasets such as Pecan Street AMI data, the MSU/ORNL power system and gas pipeline SCADA data provide useful safety-critical context. They are valuable for studying false data injection, energy-consumption manipulation, and process-aware anomaly detection. Nevertheless, their availability, scale, labeling quality, and direct applicability to adversarial ML evaluation remain more limited than general-purpose network datasets. Therefore, studies using these datasets should clearly report the attack assumptions, physical constraints, and evaluation scope.

Based on this assessment, future IIoT adversarial ML studies should avoid relying on a single dataset type. A minimal evaluation suite should include:

- At least one physical process dataset, such as SWaT, WADI, or an equivalent ICS testbed dataset, to evaluate whether adversarial perturbations remain physically plausible.
- At least one IIoT-native network dataset, such as X-IIoTID or Edge-IIoTset, to assess network-layer adversarial behavior under realistic device and traffic profiles.
- Standardized reporting of perturbation constraints, threat-model assumptions, and evaluation metrics. Studies relying exclusively on MNIST, CIFAR-10, NSL-KDD, or other non-IIoT benchmarks should explicitly acknowledge the domain gap and justify the extent to which their findings can generalize to deployed IIoT environments.

## 7. Challenges and Future Research Directions

The previous sections evaluated major attack types (Table 6, Table 7, Table 8, Table 9, Table 10, Table 11 and Table 12), defense mechanisms (Table 13, Table 14, Table 15 and Table 16), and the datasets used across the selected studies (Table 18 and Table 19). Performance comparisons across these tables allow the identification of key challenges in AML techniques for IIoT environments. Section 7.1 outlines these challenges, and Section 7.2 presents the corresponding future research directions.

### 7.1. Challenges

Lack of standardized benchmarks and evaluation protocols: The analysis of selected articles has revealed that different datasets have been employed for experimental validation. Some examples of these databases are SWaT and WADI [49,57], network intrusion benchmarks like NSL-KDD and UNSW-NB15 [60], image datasets [46], and GAN-generated [56,86]. These datasets differ in modality, structure, and the types of attacks they represent. As a result, evaluation conditions vary significantly. This makes reproducibility difficult, and a fair comparison is almost impossible.

Limited real-world validation: Only a small number of research articles evaluate their methods in real IIoT environments. Most research articles perform experiments that use simulations or synthetic data, such as federated learning scenarios [75,80] and adversarial traffic generation [56]. These experimental validations often overlook practical deployment constraints such as latency limits, hardware area restrictions, power consumption, and so on. As a result, many proposed defenses may perform well in controlled experiments but remain untested or unvalidated under real IIoT operating conditions.

Limited testing: Most studies focus on static white-box attacks such as FGSM and JSMA [46,53,61]. These attacks do not change their strategy during execution and therefore do not reflect how real adversaries adapt over time. RL-based methods [52,63] and GAN-driven attacks [56,60] show the potential for adaptive, multi-stage behavior. However, these adaptive approaches/techniques are not comprehensively validated across different datasets. In the absence of this comprehensive validation, it is difficult to evaluate the performance current defenses.

Resource-constrained Environments: Many defense techniques require heavy computation or large communication budgets. This includes GAN-based models [60,67], hybrid IDS frameworks [68], and federated learning systems [75,80]. These constraints are difficult to handle on IIoT edge devices, which often have strict limits on processing power, memory, and energy. As a result, several proposed defenses may work in controlled experimental settings but are not feasible to deploy in real resource-constrained environments.

Fragmented defense approaches: It has been observed that most defense methods target to provide remedies for specific attacks. In other words, they protect only one stage of the ML pipeline. It implies that different detection methods are required to be integrated. This integration is rare in state-of-the-art literature [58,73].

Federated learning trust issues: Federated learning studies [58,80] show that current IIoT systems remain vulnerable to malicious clients. High communication costs and heterogeneous devices further increase the complexity of the problem. All these factors make it difficult to maintain trust and stability in large, distributed IIoT deployments. Existing trust mechanisms [76] only provide partial protection without validating the solution in real environments.

Inconsistent evaluation metrics: Another observation from the analysis of selected articles is the use of heterogeneous performance metrics. These metrics include but not limited to accuracy, F1-score, AUC, perturbation norms, and reconstruction error [17,43,48,72,84]. Moreover, these performance metrics are often defined differently across selected articles. This inconsistency in performance metrics makes a fair performance comparison almost impossible.

Underexplored Hardware and Operational Constraints: A key gap in the existing literature is the lack of quantitative analysis of how IIoT-specific hardware and timing constraints shape the practicality of both attacks and defenses. Out of all studies reviewed, only [51] reports a concrete physical-timing measurement: an update time of 0.093 s and a physical manipulation range of 100–280 RPM on a motor system. This result shows that adversarial effects can be observed and measured at the level of physical actuation, not just in terms of model accuracy.

Explicit mentions of edge-deployment or real-time constraints appear in the surrounding discussion of a small number of additional studies (e.g., the studies in [15,73,87] report training-time or communication overhead figures), but these are training/communication-side costs rather than deployment-side (inference-time, on-device) measurements, and none benchmark against a specific edge-hardware class (e.g., ARM Cortex-M or equivalent). Safety-critical impact is discussed qualitatively in FDIA and process-control studies (Section 4.6 and Section 4.9) but is not quantified in terms of, for example, control-loop timing margins or fail-safe thresholds in any reviewed study. This near-total absence of deployment-side quantification—rather than a moderate gap—is itself the central finding of this subsection, and motivates the standardized reporting protocol proposed in Section 7.2, which explicitly requires computational overhead reporting on a representative edge-device class as one of four minimum metrics.

By contrast, most studies report only accuracy, F1-score, AUC, or ASR, with no information on inference latency, memory usage, or energy consumption on realistic edge hardware. Defense mechanisms that add significant computational overhead—such as GAN-based detectors (Table 16, [57,60]), federated learning with homomorphic encryption (Table 14, [87], which reports increased latency over a baseline), and ensemble or hybrid architectures (Table 15, [73], reporting a 29% increase in training time)—are rarely evaluated against the strict real-time constraints of industrial control loops. This creates a gap between reported algorithmic gains and their actual deployability on resource-limited IIoT edge devices, which this review identifies as a major barrier between laboratory prototypes and operationally relevant solutions.

### 7.2. Future Research Directions

**Standardized IIoT Adversarial Benchmark Suite:** To make future IIoT adversarial ML studies easier to compare, this review suggests using a small but more representative benchmark suite rather than relying on a single dataset. A useful baseline would combine three evaluation layers. First, a physical process dataset, such as SWaT or WADI, can be used to check whether the proposed attack remains realistic at the process level. Second, an IIoT-native network dataset, such as X-IIoTID or Edge-IIoTset, can support network-layer evaluation under more realistic device and traffic conditions. Third, a federated or non-IID split of one of these datasets can help evaluate robustness in distributed learning settings. Using this type of minimal benchmark setup would not solve all reproducibility issues, but it would make evaluation practices more consistent and would reduce part of the dataset realism gap discussed in Section 6.

**Standardized Reporting Protocol:** Future IIoT adversarial ML studies should report a small set of core metrics in a consistent way. At minimum, this should include ASR under both white-box and black-box settings, accuracy degradation under a clearly defined perturbation budget (ϵ), computational overhead on a representative edge device class, such as ARM Cortex-M or an equivalent platform, and the false positive rate on clean data. Reporting these four metrics would make it easier to compare studies even when they use different datasets, models, or attack algorithms. A simple reporting template based on these fields could also help researchers present their results more transparently and reduce the inconsistency currently observed across IIoT adversarial ML studies.

**Open-Source IIoT Adversarial Evaluation Framework:** Another important direction is the development of an open-source evaluation framework for IIoT adversarial ML. Such a framework could combine a simulated IIoT environment, for example using OpenPLC or MATLAB/Simulink-based ICS models, with existing adversarial attack libraries such as the Adversarial Robustness Toolbox (ART). This would allow researchers to test attacks and defenses under configurable physical constraints, timing limits, sensor noise, and resource profiles without always needing access to physical testbeds. Although simulation cannot fully replace real industrial validation, a shared framework would make experiments more reproducible and would provide a practical step toward closing the realism gap identified in the reviewed studies.

**Another important direction is to study adversarial attacks in wireless IIoT settings more realistically, considering how channel noise, fading, filtering, and propagation effects may weaken or change the perturbation before it reaches the deployed model.**

**Adaptive evaluation:** RL-based [52,63] and GAN-based [56,60] attacks show strong adaptive behavior. However, their evaluation across different environments is still limited. Future work should examine transferability across models, datasets, and deployment scenarios. This implies that an attack for one model can also be employed on another model. It is also important to check if a particular attack still remains effective when the dataset changes. Furthermore, attacks should be validated in different deployment settings (such as edge devices, cloud systems, and real industrial networks).

**Expanded RL-based adversarial modeling:** RL attacks [17,52,63] represent only a small portion of current AML research. Future work should expand RL-based adversarial modeling across a wider range of IIoT domains. Evaluating RL-based adversarial modeling in diverse IIoT domains can highlight new vulnerabilities. It can also show whether RL agents generalize beyond the specific systems they were trained on. Such studies are important for understanding the full impact of adaptive adversaries in real-world IIoT deployments. It should also benchmark RL attacks against other attack families.

**Lightweight, resource-aware defenses:** Many defenses introduce heavy computational demands [60,67,80]. These requirements make them difficult to deploy on IIoT edge devices, which often have limited processing power, memory, and energy. Future research should focus on designing lightweight and efficient defense mechanisms. These mechanisms should operate within strict resource budgets and still maintain acceptable robustness.

**Integrated multi-layer defense frameworks:** Current approaches test detection [64,86], prevention, and recovery [44] mechanisms separately. Future work should develop integrated frameworks that combine detection, prevention, and recovery into a single pipeline. Such frameworks can provide end-to-end resilience and reveal interactions that isolated evaluations cannot capture.

**Scalable federated learning solutions:** Federated studies [58,75,80] reveal scalability, communication, and trust challenges. Future research should focus on building robust distributed systems that operate reliably under realistic heterogeneous conditions. This includes handling diverse hardware, unstable network links, and varying data distributions across clients.

## 8. Answers to Formulated Research Questions

**RQ1: What are major AML attacks in IIoT environments, and how do they affect system performance?**

**Answer:** The analysis identifies seven attack types, discussed in Sections (Section 4.1, Section 4.2, Section 4.3, Section 4.4, Section 4.5, Section 4.6 and Section 4.7) and compared in (Table 6, Table 7, Table 8, Table 9, Table 10, Table 11 and Table 12). Looking across these categories, three broader patterns stand out.

First, the size of the performance drop does not line up neatly with how “sophisticated” an attack is. Single-step gradient attacks (Table 6) can almost completely destroy accuracy in some studies (for example, [45], from 98.7% down to 0.03%), matching or even exceeding the degradation seen for more complex RL-based attacks (Table 9). This suggests that, in the reviewed work, model fragility under white-box perturbations can be just as severe as under adaptive black-box attacks, which matters for how we weigh “attack sophistication” when prioritizing defenses.

Second, the attack types cluster by the phase they target. Gradient-based, saliency-based, GAN-based, RL-based, and FDIA attacks mainly focus on inference-time behavior, while poisoning uniquely targets the training phase. This means that a defense tailored to one phase (for example, adversarial training for inference-time robustness) offers little protection for the other (training-pipeline integrity).

Third, attention to physical constraints differs sharply across categories. FDIA (Table 11) and a subset of gradient-based studies [43,51] explicitly enforce physical process invariants, whereas most gradient-, saliency-, and GAN-based work operates on abstract feature spaces without checking whether perturbations are physically plausible. As a result, the degradation reported for these latter categories may not translate directly into real physical impact in IIoT settings.

**RQ2: What defense mechanisms have been proposed, and how effective are they in IIoT settings?**

**Answer:** The analysis of selected articles has identified four main categories of defense mechanisms against AML in IIoT environments: **(i)** adversarial training and robust learning (Section 5.1), **(ii)** run-time monitoring and anomaly detection (Section 5.2), **(iii)** hybrid or layered defenses (Section 5.3), and **(iv)** inference-time detection and classification frameworks (Section 5.4). The performance analysis of selected research articles in each defense mechanism type is presented across Table 13, Table 14, Table 15 and Table 16. Adversarial training (Table 13) provides targeted robustness gains of roughly 4–15%, but its effectiveness drops under stronger or mismatched attacks. Run-time anomaly detection (Table 14) and hybrid defenses (Table 15) achieve higher accuracy, stronger detection capability, and notable system-level improvements. Nevertheless, they introduce computational and communication overhead. Inference-time detection and classification frameworks (Table 16) deliver the highest absolute performance, but their robustness can decline under mixed or adaptive attack scenarios.

**RQ3: How do datasets differ across studies, and what are the consequences of these differences?**

**Answer:** Based on the evidence presented in Section 6 of the systematic review, dataset choice has a clear and measurable influence on both evaluation outcomes and reproducibility in AML research for IIoT, ICS, and CPS environments. The studies reviewed mainly rely on two broad dataset categories—generated datasets, which include synthetic and adversarial data, and published datasets, which are public or semi-public benchmarks—each of which provides different advantages for evaluation.

Generated datasets are widely used to test adversarial robustness under controlled and adjustable conditions. As shown in Table 18, GAN-based methods are used to generate industrial images [46], adversarial network traffic [56], and abnormal IIoT behavior [86], while gradient-based approaches such as FGSM, PGD, JSMA, and physics-constrained perturbations are used to create targeted adversarial samples for ICS and CPS signals [43,48,49]. Simulation-based datasets are also used to study routing attacks [47], RL manipulation of control systems [17], CPS security games [72], trust dynamics in federated learning [75,76,80], and power-system stability under adversarial conditions [65]. This diversity shows that generated datasets are not limited to one type of adversarial construction, but instead span GAN-based synthesis, gradient-based perturbation, and simulation-driven generation, with simulation-based approaches appearing especially important in complex CPS and IIoT settings.

Published datasets, in contrast, serve as common benchmarks that support comparability and reproducibility across studies. Table 19 shows that datasets such as SWaT, WADI, UNSW-NB15, NSL-KDD, NF-BoT-IoT, NF-ToN-IoT, DS2OS, ToN-IoT, Edge-IIoTset, X-IIoTID, and Drebin are repeatedly used in adversarial attack and defense research [55,56,60,69,73,81,82,83,87]. Image-based benchmarks such as MNIST, CIFAR-10/100, Conveyor-V3, and T-LESS are used to assess adversarial training, backdoor detection, and industrial vision robustness [46,59,67,80], while smart-grid and CPS datasets such as Electra ICS, MSU/ORNL Power System Dataset, gas pipeline SCADA, water-tank systems, and AMI data support adversarial evaluation in safety-critical domains [45,53,66,71,81,88]. More recent studies also extend evaluation to IoMT settings, where datasets such as CICIoMT2024 and ICU healthcare datasets are used to examine adversarial robustness in multi-protocol and healthcare-specific attack scenarios [70].

Across the reviewed literature, published datasets are often further augmented with adversarial samples created through gradient-based, GAN-based, RL-based, or hybrid methods to improve robustness testing [49,52,57,64,84,86]. At the same time, a relatively small group of benchmarks—especially SWaT, NSL-KDD, and UNSW-NB15—dominates the literature, while newer IIoT datasets such as Edge-IIoTset and X-IIoTID appear in a smaller but steadily growing number of studies [50,68,69,78,87]. By comparison, generated datasets tend to vary much more in structure, scale, and generation process, ranging from GAN-based synthesis to physics-constrained and simulation-driven data generation, which makes them highly flexible but less uniform across studies.

Overall, the evidence in Table 18 and Table 19 suggests that generated datasets are mainly used for controlled robustness testing and broader attack coverage, whereas published datasets provide the basis for standard evaluation and reproducibility, often with additional adversarial augmentation. However, differences in dataset size, domain, and distribution—especially with the inclusion of large-scale IIoT datasets such as X-IIoTID, modern benchmarks such as Edge-IIoTset, federated and non-IID settings such as ToN-IoT, and domain-specific IoMT healthcare data—create variability in results and make direct comparison across studies difficult.

**RQ4: What open challenges remain and what are the probable future research directions?**

**Answer:** Section 7 presents eight concrete challenges and future directions. Taken together, they reveal two deeper structural gaps.

First, there is a phase–resource mismatch. The core defenses in Section 5.1 (adversarial training) and Section 5.4 (detection-based methods) are typically evaluated without considering the latency, memory, and energy limits of IIoT edge hardware (Section 7.1, Resource-Constrained Environments). Yet the attacks they are meant to counter—RL-based and FDIA (Section 4.4 and Section 4.6)—are exactly those that matter most for real-time, physically constrained deployments. This means that, in the reviewed corpus, defense and attack research are often built on different operational assumptions, which weakens the direct applicability of reported defense gains to the most realistic IIoT threat scenarios.

Second, there is a broad lack of evaluation standardization across datasets (Section 6), metrics (Section 7.1, Inconsistent evaluation metrics), and threat models (Section 4.8 on threat-model realism). Without shared reference points along these three axes, it is hard to know whether improvements reported in one study genuinely carry over to another. Closing this gap calls not just for more datasets, but for a standardized evaluation suite and reporting protocol of the kind outlined in (Section 7.2).

### 8.1. Limitations of the SLR

Although we have adopted established systematic literature review guidelines [37], there are certain limitations or shortcomings.

- **Lack of Exhaustiveness:** Although the keywords in the search process were carefully used to cover AML research in IIoT, we do not make any claim of exhaustiveness. The field is changing quickly and involves many disciplines. Consequently, it is always difficult to complete the retrieval of research articles. It implies that title and abstract-based filtering may remove some relevant papers
- **Selective Databases:** This SLR is targeting some leading databases such as IEEE Xplore, ACM, Elsevier, Springer. While these databases cover most AML research in the IIoT domain, there is a possibility that a significant contribution in the field has been published outside these databases. However, it will not affect the overall findings of this SLR.
- **Focus on Recent Research:** This SLR targets only recent publications to identify current attacks, datasets, and defense methods. Nevertheless, this approach overlooks foundational studies that influenced modern methods. As a result, the review is more biased towards present developments than towards the field’s historical evolution.
- **Limitations of Quantitative Evaluation:** Selected research articles differ widely in their datasets, attack models, architectures, metrics, and experimental setups. Therefore, a quantitative comparison becomes difficult. Although we have tried our best to provide a quantitative comparison as much as possible, there are many places in this SLR where our synthesis focuses on qualitative patterns rather than quantitative evaluation.
- **Author-Claimed Results:** The analysis relies on the experimental results and claims presented in the selected research articles. We have not validated the claims independently.

Despite these limitations, this SLR provides an evidence-based holistic perspective of AML in the IIoT environment. It highlights the main attack types, defense approaches, and dataset practices used in recent studies.

### 8.2. On the Feasibility of Quantitative Meta-Analysis

The comparison tables in this review present author-reported performance figures side by side to summarize the range of observed effects across studies. These tables should be read as illustrative summaries rather than as inputs to a formal meta-analysis. A genuine meta-analytic synthesis requires comparability along several dimensions that are not satisfied here: studies differ in datasets (e.g., SWaT vs. NSL-KDD vs. custom ICS testbeds), model architectures (from classical ML such as RF and SVM to deep sequence models), perturbation budgets (ϵ values are inconsistently defined or omitted, as noted in Section 4.1), evaluation metrics (accuracy, F1, AUC, ASR, and reconstruction error are used interchangeably across studies), and—as detailed in Section 4.8—threat-model assumptions ranging from white-box to black-box. Aggregating, for example, an FGSM accuracy drop reported under white-box access on SWaT data with a PGD-based result reported under gray-box access on a smart-grid dataset would produce a numerically precise but substantively meaningless effect size. Before meta-analytic techniques become feasible for this domain, at minimum the following would need to be standardized:

- a shared benchmark suite (as proposed in Section 7.2);
- consistent perturbation-budget reporting;
- a common minimal metric set (e.g., ASR under both white-box and black-box conditions, as recommended in Section 7.2);
- explicit threat-model labeling of the kind introduced in Section 4.8.

Until these conditions are met, this review deliberately favors qualitative, caveated synthesis over quantitative pooling.

## 9. Conclusions

This systematic literature review has analyzed 50 carefully selected research articles on AML in IIoT environments. It has provided a holistic perspective of the domain by connecting attack types, defense methods, and databases. The articles on attack types have been further classified into seven categories. gradient-based methods (e.g., FGSM and PGD), GAN-based attacks, RL-based attacks, poisoning techniques, saliency-based approaches, false data injection attacks, and hybrid strategies. A performance comparison of selected studies in each attack category has identified its pros and cons. Gradient-based attacks remain the most commonly used due to their simplicity and reproducibility, whereas RL-based and protocol-aware attacks exhibit increased adaptability and stronger impact on detection systems across diverse data modalities. The selected articles on defense methods have been categorized into adversarial training, runtime monitoring, detection-based, and hybrid/layered approaches. A key finding is that attack effectiveness is closely tied to threat-model assumptions. Approximately half of the reviewed studies evaluate attacks under white-box conditions, while relatively few consider the black-box or gray-box settings that better reflect real-world IIoT deployments. This imbalance is most evident in gradient- and saliency-based research, whereas RL-based and FDIA studies more frequently adopt deployment-oriented assumptions. The review also shows that threat-model realism is application-dependent, as anomaly detection, process control, fault diagnosis, and closed-loop RL systems each impose different constraints on attacker capabilities.

A performance comparison of selected studies in each defense category has identified its pros and cons. Adversarial training enhances robustness against known perturbations but shows limited generalization to unseen attacks and requires high computational cost. Detection-based and hybrid methods provide broader protection but introduce additional system complexity. The analysis of datasets highlights a trade-off between realism and experimental control. Widely used benchmarks such as SWaT, WADI, and NSL-KDD offer realistic industrial scenarios but limited diversity of adversarial conditions. In contrast, synthetic and GAN-generated datasets enable flexible experimentation but reduce reproducibility and comparability. The comprehensive performance comparison of attacks, defenses, and databases has allowed the identification of challenges in the domain. Key challenges are scalability for edge deployment, insufficient evaluation under real-time and safety-critical conditions, lack of focus on adaptive adversaries, dataset fragmentation, and the omission of practical deployment constraints such as latency, energy consumption, and system overhead. Consequently, probable future directions have been proposed.

## Figures and Tables

**Table 1:** Survey and analytical studies on AML attacks/defenses in IIoT systems.

Ref.	Contributions/Insight	Identified Gaps
[25]	Identifies mismatch between academic AML research (simplified or idealized settings) and industrial security needs	Lack of a holistic or integrated perspective that can integrate attacks, defense methods, and database types
[26]	Experimental evaluation of various AML attacks for degrading model accuracy and defenses to recover performance	Do not consider industrial constraints of real IIoT environments and lacks synthesis of various databases
[27]	Presents a taxonomy to link attack mechanisms with defense strategies across ML domains	Lack of unified AML benchmarks for consistent performance evaluation and synthesis of databases
[28]	Categorizes different adversarial strategies into distinct types to show attacks behave in real systems	No standardized evaluation or benchmarks for performance comparison, lack of a holistic perspective
[29]	Provides an integrated/holistic perspective of IoT adversarial behaviors to link attacks and defenses with each other	Do not consider industrial constraints, lack of standardized benchmarks
[22]	Classifies IIoT attacks across five categories (phishing, ransomware, protocol, supply chain, system)	Low relevancy towards the AML in IIoT, lack of evaluation for defense frameworks and databases
[4]	Provides a system-level analysis of AML attacks and defense methods across industrial environments	Lack of a systematic methodology and evaluation of databases
[32]	Reveals deficiencies in ML security practices/procedures, tooling, and workflows across organizations	Lack of AML engineering guidelines and quantitative performance comparison of attacks and defense methods
[30]	Evaluates detection performance under various adversarial conditions and compares different mechanisms	Inconsistent performance evaluation, lack of standardized protocols and a holistic perspective
[31]	Integrates security, privacy, and reliability through a taxonomy to show relationship between attacks and defenses	Limited quantitative performance comparison, Lack of evaluation for defense frameworks and databases

**Table 2:** Selection and Rejection Criteria Used in the Review Process.

Selection Criteria	Rejection Criteria
Publisher: Studies must be sourced from ACM, IEEE, Elsevier, MDPI, or Springer.	Publisher: Studies published outside the targeted databases were excluded.
Subject Relevant: Must focus on AML attacks or defenses in IIoT settings.	Outside IIoT Scope: AML studies not targeting IIoT systems.
Evidence-driven Research: Selected studies must present experimental findings based on datasets, models, or simulations.	No Empirical Basis: Conceptual or purely theoretical articles without experiments, datasets, or models.
Publication Date: Selected studies must be published between 2019 and the present.	Non-Peer-Reviewed: Editorials, opinion pieces, informal articles, or unreviewed preprints.
Language: Written in English.	Multiple Studies: For multiple studies on the same topic, only one representative work is selected to avoid duplication and repetitiveness.

**Table 3:** Dimensions of Analysis Applied to the Selected IIoT Adversarial ML Studies.

Dimension	Description	Ref.	Total
Attack Types	Identifies adversarial attack types such as gradient-based, GAN-generated, RL-based, hybrid, false data injection, poisoning, and saliency-based.	[14,15,16,17,43,44,45,46,47,48,49,50,51,52,53,54,55,56,57,58,59,60,61,62,63,64,65,66]	28
Defense Mechanisms	Evaluates defense techniques: adversarial training, detection-based methods, runtime monitoring, and hybrid defenses.	[15,16,46,47,48,51,52,54,55,57,58,60,61,67,68,69,70,71,72,73,74,75,76,77,78,79,80,81,82,83,84,85,86,87,88]	35
Data Source	Describes datasets used, distinguishing between public and generated sources such as synthetic IIoT data, GAN-based signals, and simulated ICS traffic.	All Articles	50

**Table 4:** Overview of Major Adversarial Attack Mechanisms in IIoT.

Category	Main Idea	Data Type	Phase	Objectives	Advantages	Limitations
Gradient-based	Uses model gradients for minimal perturbations (FGSM, PGD, C&W).	Images, text	Inference	Misclassification, evasion	Simple; low cost; reproducible	Requires white-box access; unrealistic in deployment
GAN-generated	Synthesizes adversarial examples that resemble normal data.	Images, audio, binary	Inference, training	Misclassification, data poisoning	Realistic samples; harder to detect	High compute; unstable training
RL-based	RL agents learn sequential policies.	Sequential, time series	Inference	Disruption, policy manipulation	Adaptive; no gradient access needed	Long training time; high compute cost
FDIA	Directly manipulates sensor or measurement data streams.	Sensor, real-time	Inference/ Online	Deceive control systems, bypass detectors	No ML access needed; safety-relevant	Requires process knowledge; domain-specific
Poisoning	Inserts malicious samples into the training dataset.	Any	Training	Degrade robustness, implant back-doors	Persistent, long-lasting impact	Requires training-pipeline access
Saliency-based	Perturbs most influential input features via saliency maps.	Images, audio	Inference	Stealthy misclassifications	Stealthy; minimal perturbation	Requires feature-importance info; costly per sample
Hybrid-generated	Combines multiple approaches (e.g., gradient + GAN-based).	Multi-modal	Various	Increase transferability and evasion success	High transferability	High complexity; inherits worst-case access assumption

**Table 5:** Overview of Defense Strategies for Adversarial ML in IIoT.

Category	Description/Example Techniques	Advantages	Limitations
Adversarial Training	Training on adversarial examples or through robust optimization. Examples: PGD-based, RTCN, selective retraining, min–max optimization.	Improves known-attack robustness	High training cost; weak on unseen attacks
Detection-Based	Mechanisms that detect adversarial or abnormal inputs before model inference. Examples: statistical anomaly detectors, out-of-distribution detection, feature-integrity classifiers, input-transformation detectors.	No retraining needed; high accuracy	Unstable under mixed attacks
Runtime Anomaly	Continuous monitoring to detect inconsistencies, abnormal temporal patterns, or violations of physical invariants. Examples: temporal behavior monitors, invariant checking, sensor-fusion anomaly detection.	Continuous, real-time monitoring	High latency; not real-time-friendly
Hybrid	Multi-stage approaches combining training, detection, and monitoring mechanisms. Examples: adversarial training + runtime monitoring, reconstruction + statistical detection, layered architectures.	Comprehensive, multi-stage protection	High complexity and overhead

**Table 6:** Gradient-based/Iterative Adaptive Generation Techniques.

Ref.	Methods	Models	Mechanisms	Key Findings	Threat-Model Setting
[53]	FGSM	GB, ID3	Gradient-based perturbation (10% feature modification)	Accuracy: 89% (GB), 85% (ID3); Precision/Recall/F1 ↓ by 20–22.5%	White-box
[61]	FGSM, BIM, DeepFool	LSTM (SIEMS)	Sign-gradient, iterative, and hyperplane	Accuracy: 95–99%→30–40%; BIM most damaging	White-box
[45]	FGSM, BIM, SIGSM	ICS anomaly detection	Selective iterative gradient modulation	Baseline accuracy ≈ 98.7%; FGSM/BIM→0.03%; SIGSM→14%	White-box
[43]	VAASI (multi-norm gradient)	Industrial anomaly detectors	Adaptive $L_{1}$/$L_{2}$/$L_{\infty }$ perturbation	Precision = 0.974; Recall = 0.972; F1 = 0.973; ASR ≤ 85.5%; error = 0.028	White-box
[46]	PGD-5, PGD-20	Deep neural networks	Iterative $L_{\infty }$-bounded PGD	Accuracy: 80–90%; adversarial accuracy: 65–75%	White-box
[14]	Single-step gradient	IIS models (TEP, WM-811K)	Feature-level and smoothed-gradient	Increased transferability and transferability	Black-box/gray-box
[51]	FGSM, BIM	IIoT IDS (physical system)	Generator–discriminator perturbation	Physical manipulation: speed 100–280 RPM (motor); update time ≈ 0.093 s	Black-box/physical side-channel
[15]	FGSM, PGD, C&W, FDI	RTCN	Multi-attack adversarial training	Accuracy ↑ by 10–15%; false alarms ↓; high PGD	White-box
[62]	FGSM, PGD, JSM, C&W	CNN	Perturbation of spectrogram	Accuracy 80–90%; ASR up to 100% (PGD/JSM, ϵ≥0.3); PGD strongest	Black-box

Note: In the Results columns, ↑ indicates an increase to the reported value, ↓ indicates a decrease to the reported value, and → indicates a transition from one value to another.

**Table 7:** GAN-Based and Distribution-Bias Simulation Attacks.

Ref.	Methods	Models	Mechanisms	Key Findings	Threat-Model Setting
[57]	LSTM-GAN	CPS time-series anomaly	Fog-level reconstruction with anomaly scoring	AUC up to 0.99; EER = 0.06–0.10; ≥5.5× faster than MAD-GAN	N/A
[60]	DB-CGAN	RF, SVM, DNN	data augmentation with distribution-bias correction	UNSW-NB15: F1 = 99.52%, FAR = 2.11%; NSL-KDD: F1 = 91.32%, FAR = 2.32%	White-box
[56]	PS-WGAN + surrogate	CNN, LSTM, GRU, Kitsune	Packet-level adversarial traffic	Evasion rate ≤ 99%; transferability ≈ 30%	Gray-box/Black-box
[55]	GAN-assisted adaptive IDS	SAMKNN-based IIoT IDS	GAN-assisted scoring + STM/LTM	Accuracy ≤ 99.5%; performance degrades under mixed attacks	White-box
[47]	GAN + SVM	RPL-based IIoT routing models	GAN-assisted SVM classification	Accuracy higher than SVM baseline	White-box

**Table 8:** Poisoning Attack.

Ref.	Methods	Models	Mechanisms	Key Findings	Threat-Model Setting
[59]	DCGAN-based	CNN IDS (image-based)	GAN-generated adversarial samples	Accuracy: 0.998→0.635; with filtering: 0.967	White-box
[44]	Interpolation, back-gradient	RNN, CNN, AE	Training-time with interpolation and gradient-based	ASR: 20–60%; overfitting ↓ by 10–25%; performance increase 10–20%	White-box
[58]	Byzantine poisoning in FL	Federated CNN	Malicious updates under FL with robust aggregation	Accuracy: MNIST 91.7%, CIFAR-10 68.3%; with 20% Byzantine clients: 88.9%; communication ↓ by 35%	Gray-box

Note: Arrows indicate the direction of change in the reported metric. ↓ denotes a decrease, and → indicates a transition from one value to another.

**Table 9:** Reinforcement Learning-Based Generation Attacks.

Ref.	Methods	Models	Mechanisms	Key Findings	Threat-Model Setting
[63]	A2C RL-based	ICS with rule-based IDS	RL agent learns sequential sensor-spoofing policies	Detection rate: 100%→0% after 600 episodes	Black-box
[52]	PPO attacker-defender	Multivariate IIoT time series	Adaptive perturbation generation via RL policy	F1-score = 0.825; +14.7% vs. VAE (0.724) and OmniAnomaly (0.676)	Gray-box
[17]	IRL-based adversarial perturbation	IIoT control agents	Reward-function using inverse RL approximation	Higher vulnerability; performance–security trade-off observed	Gray-box

Note: In the Results columns, → indicates a transition from one value to another.

**Table 10:** Saliency-Based Attacks.

Ref.	Methods	Models	Mechanisms	Key Findings	Threat-Model Setting
[48]	JSMA	RF, J48 (ICS IDS)	Perturbation of input features	Accuracy drop: RF ↓ by 6 points; J48 ↓ by 11 points	Gray-box
[49]	JSMA	CART, RF, IDS	Perturbation on sensor and actuator	Accuracy ≈ 95%	White-box
[54]	Jacobian-based attack	KBL	Perturbation with selective retraining	Accuracy ↑ by 6%; AUC = 0.958; F1 and G-mean ↑; overfitting when >65% adversarial samples	white-box
[62]	JSM (FGSM, PGD, C&W)	CNN	Perturbation of spectrogram	Accuracy: 80–90%; JSM ASR up to 100%	Black-box

Note: Arrows indicate the direction of change in the reported metric. ↑ denotes an increase, ↓ denotes a decrease.

**Table 11:** False Data Injection Attacks.

Ref.	Methods	Models	Mechanisms	Key Findings	Threat-Model Setting
[16]	FDIA (Dual-VAE, GRU)	Smart grid FDIA detection	Adversarial generation using VAEs	Higher AUC-ROC and AUC-PR	Gray-box

**Table 12:** Hybrid-Generated Attacks.

Ref.	Methods	Models	Mechanisms	Key Findings	Threat-Model Setting
[50]	FGSM, PGD	IDS (X-IIoTID)	Diverse perturbation patterns	Attack ↑ by 36%; F1 ↓ by 61%	Black-box
[64]	Temporal perturbation	LSTM Autoencoder	Augmentation	Accuracy ≤96.83%; F1-score ≥94% across datasets	White-box
[65]	PCAA	RF-based DTSA; NN models	Optimization-based/ physics-constrained	RF: Accuracy 0.98→0.38; Recall 1.00→0.40; F1-score 0.99→0.55 transfer accuracy: 0.75 (DNN), 0.65 (KNN), 0.85 (SVM)	White-box/Black-box
[66]	Opt. Attack + GAN attack	Substitute MLP; target stacked IDS using RF, Extra Trees, AdaBoost, GBDT, and XGBoost	Protocol-constrained evasion with payload preservation	Target IDS: P = 99.40%, R = 99.39%, F1 = 99.39%; Opt. ASR: injection = 20.958%, function-code = 81.201%, reconnaissance = 100%; GAN.reconnaissance = 100%	Black-box

Note: Arrows indicate the direction of change in the reported metric. ↑ denotes an increase, ↓ denotes a decrease, and → indicates a transition from one value to another.

**Table 13:** Adversarial Training Defense Mechanism.

Ref.	Defense	Model	Attack	Performance	Dataset
[48]	Adversarial Training	RF, J48	JSMA	RF ↑ 6%; J48 ↑ 11%	ICS power-system
[68]	EIFDAA Training	CNN, GRU, DT, HyDL, SVM, RF, KNN	FGSM, BIM, PGD, WGAN-GP	misclass > 94%; PGD: HyDL 10.36→0%; DT 14.89→13.43%	X-IIoTID
[71]	Robust Regression	Time-series regression	FGAV, RSL, evasion	30% fewer attacks; F1 ↑ up to 61%	Pecan Street AMI
[51]	GAN-Based Learning	GD, DNN, IDS	FGSM, PGD	Acc 93.7→97.9%; F1 ↑ 4%	ToN-IoT
[54]	Selective Retraining	DNN, RF, SVM, KBL, Bayesian	White-box JSMA	Acc 98.3→71.0%; KBL 86.08%, AUC 0.958; random 80.69%, AUC 0.884	Android malware
[74]	CWT-CNN	Gaussian noise	FDI + FGSM	Acc 94.06%	Smart-meter dataset
[15]	PGD-Based Training	RTCN (dilated causal TCN)	FDI, FGSM, PGD, C&W, noise	Robust accuracy ↑ 10–15%	IIoT sensor-fault dataset
[16]	AT-GVAE	Dual VAE (GRU + attention)	FDI attacks	AUC-ROC/AUC-PR ↑ vs. GRU/VAE; better localization	Smart-grid sequences
[69]	Ensemble Training	BiGRU, LSTM, CGAN, SHAP	Auto-PGD, Square, CW, DeepFool	Acc > 99%; ADR < 0.15%; <140 MB	Edge-IIoTset; CIC-IoT2023

Note: Arrows indicate the direction of change in the reported metric. ↑ denotes an increase, and → indicates a transition from one value to another.

**Table 14:** Run-time/Anomaly Detection Mechanisms.

Ref.	Defense Type	Core Mechanism	Attack Type	Performance
[78]	GAT-Based APT	Graph Attention Network with masked self-attention	Multi-stage intrusions	Accuracy: 96.97% (DAPT2020), 95.97% (Edge IIoT); prediction time 20–21 s
[87]	Federated Anomaly Detection	homomorphic encryption, differential privacy, adaptive thresholds	Poisoning, model inversion, PGD perturbations	F1 = 88.5%; FPR = 2.7%; adversarial success = 8.3%; communication ↓ 53%; convergence ↑ 23%
[88]	Reactive autoencoder-based	RADIANT: multi-autoencoder reconstruction-error defense	Evasion attack (HopSkipJump, ZOO)	HSJ: F1 = 0.914, FPR = 0.012; ZOO: F1 = 0.859, FPR = 0.014

Note: Arrows indicate the direction of change in the reported metric. ↑ denotes an increase, ↓ denotes a decrease.

**Table 15:** Hybrid/Layered Defense Mechanisms.

Ref.	Defense Type	Core Mechanism	Attack Type	Performance
[58]	Hybrid Fault-Tolerant FL	Aggregation, anomaly detection, blockchain, continual FL	Byzantine faults, poisoning, gradient manipulation	MNIST: 91.7% vs. 85.2%; CIFAR-10: 68.3% vs. 61.5%; 20% Byzantine: 88.9%
[61]	Adv. Training + Distillation	Retrained LSTM SIEM with softened-output distillation	FGSM, BIM, DeepFool	Accuracy: 30–40%→>80% under attack
[72]	Game-Theoretic CPS Defense	Adversarial RL attacker + MDP-based defender (Nash equilibrium)	Sequential CPS attacks	Time-to-breach ↑ ∼25%; convergence ≤10 iterations
[77]	Privacy-Preserving Security	Key management, encryption, anomaly detection, frequency hopping	FDI, jamming, replay, eavesdropping	Comm. overhead ≤20kB; re-encryption <250ms; HE <60ms
[80]	Privacy-Preserving FL + Blockchain	DNN with DPSGD under FedAvg; blockchain aggregation	Poisoning, gradient inference	Accuracy ↑3.7%; latency ↓28%; energy ↓21%
[73]	Hybrid FL + GAN	TSRN-GAN synthetic data, adaptive aggregation, quality filtering	Synthetic adversarial attacks	Accuracy 96.8%; F1 ≤95.1%; +6.8% gain; convergence ↑34%; comm. rounds ↓42%
[46]	Bio-Inspired Discriminator + Adv. Training	V1 cortex front-end (Gabor filter bank) fused with GAN discriminator (VOneGAN, V1+ResNet-50); adversarial fine-tuning	PGD-20; PGD-5 $\ell_{\infty }$	Conveyor-V3 (PGD-20): baseline 97%→0% at ϵ=0.01; V1+ResNet-50 94%→50% at ϵ=0.01 and 27% at ϵ=0.02. CIFAR-10 (PGD-5): +7–9 pts over baseline at ϵ=0.05–0.10.

Note: Arrows indicate the direction of change in the reported metric. ↑ denotes an increase, ↓ denotes a decrease, and → indicates a transition from one value to another.

**Table 16:** Detection-Based Defense Mechanisms.

Ref.	Technique	Model/Algorithm	Attack Type	Metrics	Dataset
[67]	STRIP + CycleGAN	CNN microservice + CycleGAN	Backdoor, poisoning	ASR 98–100%→0–3%; clean acc ↓ 1–3%	Custom IIoT
[82]	Multi-View Ensemble	AutoML SVM ensemble	Evasion, fabrication	Acc 91%; Prec 0.94; Rec 0.95	Drebin malware
[47]	GAN–SVM Routing	GAN feature generation + SVM	RPL routing attacks	Higher accuracy than SVM baseline	Simulated RPL IIoT
[52]	RAAD (RL-Based)	PPO attacker–defender agents	Adversarial time-series anomalies	F1 ↑ 14.7% (0.825); VAE 0.724; OmniAnomaly 0.676	SWaT, HAI, NSL-KDD
[79]	SHAP-Based	LSTM + SHAP + PCA	Modified BIM	F1 = 0.94 (TEP), 0.99 (SWaT)	TEP, SWaT
[81]	Federated	AE + FedAvg + FCN + JAP	DoS, DDoS, data theft	F1 = 0.92–0.98; Acc = 95.84% (Gas), 90.73% (Water)	SCADA gas, water tank
[83]	DRL–GAN IDS	Distributional RL + WGAN-GP	Multi-class IIoT IDS	Acc 99.02%; Prec 99.62%; F1 99.26%	DS2OS
[55]	Online Adaptive	SAMKNN + STM/ LTM + GAN	Zero-day attacks	NF-BoT-IoT: Acc 98.09%; NF-ToN-IoT: Acc 96.56%,	NF-BoT-IoT, NF-ToN-IoT
[57]	GAN	LSTM+ encoder	Zero-day	AUC 0.92–0.99; EER 0.06–0.10	SWaT, WADI, NSL-KDD
[60]	GAN	DB-CGAN + AE	adversarial perturbations	F1 = 81.6–99.5%; FAR = 1.11–4.01%	NSL-KDD, UNSW-NB15
[75]	Trust-Based	EMA + TOPSIS	Label flipping	Accuracy/AUC = ±1–2%	Federated IIoT
[76]	Evidence-Fusion	Dempster–Shafer + Markov trust + MADM	Data falsification	Stable under ±15% risk variation	IoT ecosystem
[84]	GAN-Based Detection	CCGAN + WHO + AES	DoS, Probe, R2L, U2R	Accuracy 99.21%	NSL-KDD
[85]	GAN-Based IIoT Detection	GAN + PCA + Z-score	Multi-attack (DS2OS)	Best: Prec/Rec/F1 ≤ 1.0; Worst: Prec 0.27, Rec 0.67, F1 0.77	DS2OS
[86]	Memory-Augmented GAN	GAN +AE	Binary anomaly detection	AUC 0.946; Acc 88.50%; F1 0.901	NSL-KDD
[70]	MARD Detection	Teacher–student NN + GMR + MMI + TinyML	PGD, BIM, DeepFool, DT	Robustness 99.56% (ICU); Acc > 99% across attacks	CICIoMT2024, ICU Healthcare

Note: Arrows indicate the direction of change in the reported metric. ↑ denotes an increase, ↓ denotes a decrease, and → indicates a transition from one value to another.

**Table 17:** Cross-Method Comparison of Defense Categories.

Defense Category	Robustness	Computational Overhead	Applicability to Constrained IIoT Edge Devices
Adversarial Training	Moderate, targeted gains (4–15% robust accuracy); degrades against unseen or stronger attacks	High training cost; low inference cost once deployed	Feasible if training is performed offline or centrally
Detection-Based	Highest absolute performance reported (often >99% accuracy, F1 up to 99.5%); least stable under mixed attacks	Moderate to high, especially for GAN-based detectors	Moderate; lightweight statistical detectors are edge-feasible, GAN-based ones generally are not
Runtime Anomaly Monitoring	Strong detection capability with low false-positive rates; less tested against adaptive attacks	High latency in some methods (20–21 s); encryption-based variants add further overhead	Limited for latency-sensitive control loops; better suited to offline or batch monitoring
Hybrid/Layered	Strongest overall protection, combining multiple mechanisms	Highest overall overhead (added training time, communication, complexity)	Most difficult to deploy at the edge without dedicated resources

**Table 18:** Synthetic Dataset Categories and Their Usage.

Category/Dataset	Generation Method	Models Used	Context
**GAN-Based Approaches**			
GAN visual data	VOneGAN	V1+CNN, AC-GAN, RobGAN, ResNet-50	[46]: Synthetic industrial images; PGD robustness.
GAN adversarial traffic	PS-WGAN	Surrogate classifier, NIDS	[56]: Adversarial IIoT traffic for NIDS evasion.
GAN abnormal traffic	Memory-augmented GAN	MeAEG-Net	[86]: Abnormal traffic via reconstruction error.
GAN minority traffic	DB-CGAN	RF, SVM, DNN	[60]: Minority-class IIoT traffic.
ICS network packets	Opt. attacks + GAN	MLP; RF, Extra Trees, AdaBoost, GBDT, XGBoost	[66]: Evasive injection/function code/ reconnaissance attacks vs. ICS IDS; up to 100% evasion
CycleGAN samples	Image translation	CNN, CycleGAN	[67]: Synthetic poisoned images.
**Gradient-Based Adversarial Methods**			
JSMA adversarial data	CleverHans (MLP)	RF, J48, MLP	[48,49]: JSMA perturbations for ICS/SWaT.
FDI smart grid data	FDI perturbation	CNN, ANN, LR, LDA, SVC	[16,74]: Synthetic FDI attacks.
Physics-constrained ICS data	Gradient + constraints	VAASI (AE + gradient)	[43]: Physically consistent ICS signals.
Hydraulic sensor data	FGSM, PGD, C&W	LDA, SVM, CNN, RNN, LSTM, TCN, RTCN	[15]: Perturbed hydraulic data.
Pipeline time-series	Signal perturbation	LSTM Autoencoder	[64]: Synthetic pipeline anomalies.
**Simulation-Driven Generation**			
Simulated IIoT/CPS traffic	RPL routing simulator	GAN-C, SVM	[47]: Routing attacks for low-power IIoT.
DRL control data	DRL simulation	IRL attacker	[17]: Poisoned IIoT control trajectories.
CPS attack-graph data	Hybrid attack-graph simulation	Actor–Critic RL, BO, MDP	[72]: CPS defense with simulated adversary.
Synthetic + real ICS data	Poisoning (interpolation + back-gradient)	Multiple NN models	[44]: Mixed ICS + TEP simulation.
simulation	FL simulation	FedAvg, TOPSIS+EMA	[75]: MEC-enabled FL with adversarial clients.
FL + blockchain simulation	DP-FL + blockchain	NN (DPSGD)	[80]: Simulated IIoT with DP, FL, blockchain.
Power system simulation	IEEE 68-bus transient simulation	DTSA, RF, SVM, DNN, KNN	[65]: Physics-based perturbations.
Microgrid PV system	FGSM, BIM, DeepFool	LSTM, DNN I-EMS	[61]: Perturbed microgrid sensor/weather data.
IoT trust simulation	Markov-chain risk simulation	Statistical trust model, TOPSIS	[76]: Synthetic trust scores.

**Table 19:** Published Datasets and Their Usage.

Dataset	Type	References, Models, Context
MNIST/Conveyor-V3/CIFAR10/CIFAR100	Image	[67]: STRIP + CycleGAN for backdoor defense. [80]: CNN + FL + DP for privacy. [46]: VOneGAN/AC-GAN/RobGAN for synthetic data.
Electra ICS	ICS	[45]: FGSM/BIM/SIGSM for ICS evasion.
TEP/WM-811K Wafer Map	Industrial	[14]: SNGD + ML models for fault detection.
SWaT	CPS	[49]: CART/RF/GB for JSMA IDS. [57]: FID-GAN for fog IDS. [52]: RAAD PPO for CPS IDS. [64]: LSTM Autoencoder for anomalies.
WADI	CPS	[57]: FID-GAN for fog IDS. [64]: LSTM Autoencoder for anomalies.
X-IIoTID	IIoT IDS	[50]: HD + 1D-CNN for adversarial samples. [68]: CNN/GRU/RF/SVM/HyDL under FGSM/PGD/DeepFool/WGAN-GP. [87]: Federated anomaly detection.
Edge-IIoTset	IIoT IDS	[78]: Node2Vec for APT detection. [69]: CGAN + adversarial training for IDS robustness.
UNSW-NB15	NIDS	[60]: DB-CGAN for imbalance. [52]: RAAD PPO for adversarial IDS.
NSL-KDD	NIDS	[57]: FID-GAN for reconstruction IDS. [60]: DB-CGAN for imbalance. [52]: PPO RL IDS. [84]: CCGAN + AES for smart city IDS. [86]: MeAEG-Net for anomaly detection.
CIC-IDS2017/CSE-CIC-IDS2018	NIDS	[63]: CNN + FGSM/PGD training. [73]: FL + GAN for edge IDS.
CIC-IoT2023/NF-BoT-IoT/NF-ToN-IoT/Kitsune	IoT/NetFlow	[55]: SAMKNN + GAN for zero-day detection. [56]: Adversarial traffic + defenses. [69]: CGAN + adversarial training.
ToN-IoT	IoT/IIoT	[73]: FL + TSRN-GAN for non-IID attack detection.
Pecan Street AMI	Smart Grid	[71]: Regression + robust loss for poisoning/evasion.
Hydraulic System (UCI)	Industrial Time Series	[15]: CNN/LSTM/TCN for condition monitoring.
ICS Time-Series Dataset	ICS	[79]: LSTM + BIM + SHAP for interpretable detection.
Gas Pipeline SCADA/Water Tank (MSU)	ICS Testbed	[81]: Federated AE + FCN for privacy-preserving IDS.
Power Distribution ICS Testbed	Semi-public ICS	[53]: GB/ID3 + FGSM for AML impact.
Android Malware/Drebin	Mobile Security	[54]: DNN + JSMA for malware detection. [82]: Ensemble ML for feature manipulation.
DAPT2020 (APT Malware)	Malware	[78]: Node2Vec for APT detection.
Audio Dataset	Acoustic	[62]: CNN + FGSM/PGD/C&W for audio attacks.
DS2OS	Synthetic IIoT	[85]: GAN + PCA for anomaly detection. [83]: DRL-GAN for attack detection.
HAI Dataset	CPS	[52]: PPO RL for anomaly detection.
CICIoMT2024	IoMT	[70]: Multi-attack IoMT IDS.
MSU/ORNL Power System Dataset	Published ICS dataset	[88]; RADIANT IDS with RF classifier; includes PMU, Snort, control panel, and relay logs; HopSkipJump and ZOO were used to generate adversarial samples for evaluation.
S7comm (water tank), Modbus (gas pipeline), IEC 104 (honeypot)	ICS testbed	[66]: Stacked IDS (RF, Extra Trees, AdaBoost, GBDT → XGBoost); adversarial packets validated by water level, PLC state, and traffic behavior.

## Data Availability

No new data were created or analyzed in this study. Data sharing is not applicable.

## Abbreviations

RL	Reinforcement Learning
FDIA	False Data Injection Attack
FGSM	Fast Gradient Sign Method
PGD	Projected Gradient Descent
BIM	Basic Iterative Method
SIGSM	Sign Iterative Gradient Sign Method
VAASI	Variational Adversarial Attack with Statistical Inference
SIEM	Security Information and Event Management
ICS	Industrial Control Systems
TCN	Temporal Convolutional Network
ASR	Attack Success Rate
CPS	Cyber–Physical Systems
AUC	Area Under the Curve
EER	Equal Error Rate
GRU	Gated Recurrent Unit
RPL	Routing Protocol for Low-Power and Lossy Networks
SVM	Support Vector Machine
FL	Federated Learning
DCGAN	Deep Convolutional GAN
RNN	Recurrent Neural Network
AE	Autoencoder
A2C	Advantage Actor–Critic
PPO	Proximal Policy Optimization
VAE	Variational Autoencoder
JSMA	Jacobian-based Saliency Map Attack
RF	Random Forest
AUC-ROC	ROC Area Under Curve
AUC-PR	PR Area Under Curve
PCAA	Physics-Constrained Adversarial Attack
TSA	Transient Stability Assessment
DNN	Deep Neural Network
KNN	K-Nearest Neighbors
CWT	Continuous Wavelet Transform
DT	Decision Tree
ADR	Attack Detection Rate
APT	Advanced Persistent Threat
ANN	Artificial Neural Network
FPR	False Positive Rate
STRIP	Strong Intentional Perturbation
RAAD	RL-based Adversarial Anomaly Detection
SAMKNN	Self-Adjusting Memory KNN
MARD	Multi-Agent Reinforcement Detection
ICU	Intensive Care Unit
SHAP	SHapley Additive exPlanations
DRL	Deep Reinforcement Learning
CCGAN	Conditional CycleGAN
TEP	Tennessee Eastman Process
SWaT	Secure Water Treatment
WADI	Water Distribution
NIDS	Network Intrusion Detection System
AMI	Advanced Metering Infrastructure
IoMT	Internet of Medical Things
